# Phylogeny and Morphology of Novel Species and New Collections Related to *Sarcoscyphaceae* (*Pezizales*, *Ascomycota*) from Southwestern China and Thailand

**DOI:** 10.3390/biology12010130

**Published:** 2023-01-13

**Authors:** Ming Zeng, Eleni Gentekaki, Kevin D. Hyde, Qi Zhao, Neven Matočec, Ivana Kušan

**Affiliations:** 1 School of Science, Mae Fah Luang University, Chiang Rai 57100, Thailand; 2 Center of Excellence in Fungal Research, Mae Fah Luang University, Chiang Rai 57100, Thailand; 3 Yunnan Key Laboratory of Fungal Diversity and Green Development, Key Laboratory for Plant Diversity and Biogeography of East Asia, Kunming Institute of Botany, Chinese Academy of Sciences, Kunming 650201, China; 4 Innovative Institute of Plant Health, Zhongkai University of Agriculture and Engineering, Guangzhou 510225, China; 5 Institute of Applied Fungi, Southwest Forestry University, Kunming 650224, China; 6 Laboratory for Biological Diversity, Ruđer Bošković Institute, Bijenička Cesta 54, 10000 Zagreb, Croatia

**Keywords:** 3 new species, 1 new record, 1 synonym, taxonomy, multigene analysis

## Abstract

**Simple Summary:**

Species of *Sarcoscyphaceae* are saprobic on branches, stumps, trunks, or twigs. The majority of members in this family are widespread in tropical areas, with only a fraction of the known species found in temperate areas. All species have typical disc- or cup-shaped fruiting bodies in a variety of colours ranging from white, grey, orange, red to brown. A high diversity of *Sarcoscyphaceae* has been reported in southwestern China and Thailand. In this study, we provide redescriptions of five known species and establish three new species in *Sarcoscyphaceae* from these regions based on morphology and phylogeny. We also propose an amendment for *Phillipsia gelatinosa*. *Cookeina sinensis*, a common species in China, is reported from Thailand for the first time.

**Abstract:**

*Sarcoscyphaceae* (*Pezizales*) is distinguished by small to large, vividly-coloured sessile to stipitate apothecia, plurinucleate and pigmented paraphyses, operculate asci with thick walls, and plurinucleate, uniguttulate to multiguttulate ascospores with smooth walls or ornamentations. We collected more than 40 *Sarcoscyphaceae* specimens from dead twigs or wood. Based on morphology and phylogeny, these species belong to *Cookeina*, *Nanoscypha*, *Phillipsia*, *Pithya*, and *Sarcoscypha*. Among these, we introduce three new species–*Nanoscypha aequispora*, *Pithya villosa*, and *Sarcoscypha longitudinalis*. Phylogenetic analyses based on ITS, LSU, SSU, *rpb2*, and *tef-1α* gene regions indicate the relationships of these species within *Sarcoscyphaceae*. Meanwhile, we propose *Ph. gelatinosa* as a synonym of *Ph. domingensis*. One new record of *C. sinensis* is reported from Thailand.

## 1. Introduction

*Sarcoscyphaceae* comprises discomycetous fungi that occur abundantly in tropical areas but are also found in temperate regions [1,2,3]. Le Gal [4] improperly introduced *Sarcoscyphaceae* without supplying a Latin description. Eckblad [5] provided a legitimate description according to proper nomenclature standards. However, transfer of the type genus of *Sarcosomataceae* to *Sarcoscyphaceae* caused a long-term conceptual confusion between these two families [4,5,6,7]. It was not until clarification by Korf [8] that *Sarcoscyphaceae* had a clear concept. *Sarcoscyphaceae* has typical apothecia of Pezizomycetes (commonly referred to as cup fungi) and comprises one of the few families with no records of hypogeous taxa [2]. The ascal apical apparatus, one of the most distinctive characters of the whole group, was a point of confusion for decades. Chadefaud [9] and Le Gal [4] considered some ascal apical structures that were apparently hypothetical structures and/or the artifacts that occurred during the process of material fixation. They proposed that these structures represented a transition between an inoperculate apical ring and a true operculum. They called that kind of ascus ‘paraoperculate’ or ‘suboperculate’. They proposed that this ascus represented an intermediate stage towards the evolution of operculate forms. Eckblad [5,10], van Brummelen [11,12], Samuelson [13], and Samuelson et al. [14] showed that the apical apparatus type of *Sarcoscypha* (Fr.) Boud. is in fact operculate and by no means a transitional form between inoperculate ascus apical ring and true pezizalean operculum. This has subsequently been supported in numerous phylogenetic analyses. *Sarcoscyphaceae* is characterized by vividly-coloured, sessile to stipitate apothecia, pigmented paraphyses containing carotenoids, thick-walled asci equipped with narrow and thick lenticular operculum encircled by a subapical markedly thickened wall (suboperculum), and uniguttulate to multiguttulate ascospores with smooth walls or cyanophobic lateral striation/reticulation [2,6]. There are only a few reports of anamorphs in this family. Pfister [2] and Ekanayaka et al. [3] have provided the most recent summaries. There are 13 genera in the family, namely, *Aurophora* Rifai, *Cookeina* Kuntze, *Geodina* Denison, *Kompsoscypha* Pfister, *Microstoma* Bernstein, *Nanoscypha* Denison, *Phillipsia* Berk., *Pithya* Fuckel, *Pseudopithyella* Seaver, *Rickiella* Syd. & P. Syd. ex Rick, *Sarcoscypha*, *Thindia* Korf & Waraitch and *Wynnea* Berk. & M.A. Curtis, with a total of 83 estimated species in this family [15,16]. Within *Sarcoscyphaceae*, several species have been used as food and medicine. For example, *Cookeina speciosa* (Fr.) Dennis and *C. tricholoma* (Mont.) Kuntze are treated as edible fungi in Mexico, while there are also records of their use in treating ear infections in Cameroon [17,18,19]. The Scarlet elf cup, *Sarcoscypha coccinea* (Jacq.) Lambotte, has also been said to be edible [20].

The earliest phylogenetic study of *Sarcoscyphaceae* traces to Harrington et al. [21], who used the nuclear small subunit rRNA (SSU) gene region to reconstruct the phylogeny of *Pezizales*. Phylogenetic analysis of nine sequences involving nine genera revealed the monophyly of *Sarcoscyphaceae* and its placement in *Pezizales* [21]. Romero et al. [15] added molecular data for a known species of *Rickiella* and explored phylogenetic relationships within *Sarcoscyphaceae* based on nuclear large subunit rRNA (LSU) and SSU rDNA sequences. Angelini et al. [22] introduced a new species of *Geodina* based on morphology and phylogenetic analysis using LSU, but it was later shown to be a synonym of the type species by Pfister et al. [23]. Pfister et al. [23] proposed the new family Wynneaceae, which contained *Geodina* and *Wynnea*, thus separating these taxa from *Sarcoscyphaceae* based on phylogenetic analyses of four genetic markers and morphology. The two genera also exhibit morphological characteristics and habitat preferences that distinguish them from other genera of *Sarcoscyphaceae*. However, gene regions from different strains were combined to represent certain taxa, which were then used for phylogenetic inference. Hence, establishing a new family should be put on hold until genetic markers from the taxa of interest are available to avoid confusion [23]. Unfortunately, *Aurophora* and *Thindia* still lack molecular data, and therefore their phylogenetic placement currently remains unknown.

In this study, we collected 45 specimens related to *Sarcoscyphaceae* from southwestern China and Thailand. Through morphological examinations and phylogenetic inferences based on ITS, LSU, SSU, *rpb2*, and *tef-1α*, we introduce three new species within *Nanoscypha*, *Pithya* and *Sarcoscypha*. *Cookeina* collections separate into four distinct clades, which mainly belong to four species. Following re-examination of the type specimen of *Ph. gelatinosa* Ekanayaka, Q. Zhao & K.D. Hyde, we suggest that *Ph. domingensis* (Berk.) Berk. ex Denison takes precedence over *Ph. gelatinosa*.

## 2. Materials and Methods

### 2.1. Sample Collection, Morphological Examination, and Deposition

All specimens were collected from dead wood or twigs from southwestern China and southern Thailand. Fresh specimens were dried in a dehydrator at 25–30 °C shortly after collection to prevent decay. At the same time, a small amount of tissue material from each fresh sample was put into a labeled zipper sealed bag containing allochroic silica gel for moisture absorption to be used for molecular work. All materials were brought back to the laboratory for morphological and molecular studies. Four herbarium specimens labeled *Phillipsia gelatinosa* (MFLU 15-2360, MFLU 16-2956, MFLU 16-2992) and *Phillipsia subpurpurea* (MFLU 16-0612) were borrowed from the Herbarium of Mae Fah Luang University (MFLU) for further morphological investigation.

Documentations, descriptions, and measurements of macroscopic features, including colour, shape, and size of ascomata, were recorded before fresh specimens were processed. Morphological features indistinguishable to the naked eye were photographed using a Leica M125 C stereo microscope (Leica Microsystems GmbH, Wetzlar, Germany). Colour descriptions follow RAL Colour Chart [24]. Hand sections of ascomata were performed using a Motic SMZ-168 stereoscope (Speed Fair Co., Ltd., Hong Kong, China). Dried specimens were rehydrated in distilled water, or treated with 5% or 10% KOH solution, and stained with Cotton Blue (CB), Congo Red (CR), and Melzer’s reagent (MLZ) solutions. A Nikon Eclipse Ni compound microscope with a Nikon DS-Ri2 camera (Nikon Instruments Inc., Tokyo, Japan) were used for microscopic photography. The Tarosoft^®^ Image Frame Work program v.0.9.7 (Tarosoft, Nontha Buri, Thailand) was used for measuring microscopical features. The measured number of ascospores (n), ascomata (m) and specimens (p) was denoted as [n/m/p]. Minimal (a–) and maximal (–b) values of length and width of ascospores, the 90% confidence interval (b–c) were provided as (a–)b–c(–d). Ascospore length/width ratio was referred to as Q, and **Q** values (average Q ± standard deviation) were provided to indicate the ascospore shape [25]. Photoplates were assembled using Adobe Photoshop CS6 (Adobe Systems, San Jose, CA, USA).

Specimens were deposited at the Herbarium of Mae Fah Luang University (MFLU) and Herbarium of Cryptogams Kunming Institute of Botany Academia Sinica (HKAS). Facesoffungi and Index Fungorum numbers were obtained as in Jayasiri et al. [26] and Index Fungorum [27]. The newly-generated data were added to the Greater Mekong Subregion webpage [28].

### 2.2. DNA Extraction, PCR Amplification and Sequencing

DNA was extracted from treated ascomata tissues (see Section 2.1) using the *Trelief*^TM^ Plant Genomic DNA Extraction Kit (Tsingke Biotechnology Co., Ltd., Beijing, China). Polymerase chain reaction (PCR) was used to amplify the internal transcribed spacer (ITS), the large subunit rRNA (LSU), the small subunit rRNA (SSU), the second-largest subunit of RNA polymerase II (*rpb2*), and the translation elongation factor-1 alpha (*tef-1α*). Amplifications of ITS, LSU, SSU, *rpb2* and *tef-1α* loci were performed using primer pairs ITS5/ITS4 [29], LR0R/LR5 [30], NS1/NS4 [29], fRPB2-5f/fRPB2-7cR [31], and 983F/2218R [32], respectively. The total volume of each PCR reaction mixture was 25 μL containing 9.5 μL sterile deionized water, 12.5 μL of 2× Power Taq PCR MasterMix, 1 μL of each primer (10 μM stock) and 1 μL DNA template. Amplifications were carried out using an Applied Biosystems 2720 thermocycler (Foster City, CA, USA). The cycling conditions of PCR amplification included initial denaturation at 94 °C for 5 min, followed by 35 cycles (ITS, LSU, SSU and *tef-1α*) or 40 cycles (*rpb2*) of: denaturation at 94 °C for 50 s, annealing at 56 °C for 50 s (ITS, LSU, SSU and *tef-1α*) or 55 °C for 2 min (*rpb2*), extension at 72 °C for 1 min, and a final extension at 72 °C for 10 min. The obtained PCR products were purified and sequenced by Tsingke Company, Beijing, P.R. China.

### 2.3. Phylogenetic Analysis

The raw sequences were assembled using DNASTAR Lasergene SeqMan Pro v.7.1.0 (44.1) (DNAStar Inc., Madison, WI, USA). Sequences spanning the spectrum of available diversity of *Sarcoscyphaceae* were downloaded from GenBank (Table 1). Individual sequence datasets of five gene regions were aligned using MAFFT v.7 available online [33]. All datasets were trimmed by TrimAl v.1.2 with the user-defined option (ITS: 0.9 value for gap threshold; LSU and SSU: 0.5 value for gap threshold) and gappyout option (*rpb2* and *tef-1α*) [34]. Individual datasets were used to construct phylogenetic trees for each genetic marker to assess the topological congruence of the five datasets (data not shown). A dataset combining all five genetic markers was assembled into a matrix using Sequence Matrix v.1.8 [35]. AliView v.1.19-betalk was used to convert file format [36].

Maximum likelihood (ML) and Bayesian inference (BI) analyses were carried out on CIPRES Science Gateway v.3.3 platform [37] using RAxML-HPC2 v.8.2.12 [38] and MrBayes v.3.2.7a on XSEDE [39,40]. Maximum likelihood analysis was performed using the GTR + I + G substitution model with 1000 rapid bootstrap replicates. For BI analysis, GTR + I + G substitution was selected as best-fit model of evolution for each gene using MrModeltest v.2.3 [41] as performed by MrMTgui [42] based on the Akaike information criterion [43]. Markov Chain Monte Carlo Sampling (MCMC) was used to calculate posterior probabilities (PP) [39,44]. Two runs comprising of six simultaneous Markov Chains each were run for 635,000 generations for ITS tree and 9000 generations for combined gene tree, and trees were sampled every 100th generation [45]. The first 25% of the trees were discarded as burn-in and analysis was stopped when the standard deviation of split frequencies reached 0.01.

Phylogenetic trees were viewed in FigTree v.1.4.2 [46] and edited using Adobe Illustrator CS5 (Adobe Systems, San Jose, CA, USA).

**Table 1:** Sequences used in this study.

Species Name	Country	Voucher/Strain Number	ITS	LSU	SSU	*rpb2*	*tef-1α*	References
*Chorioactis geaster* ♦	USA	ZZ2 FH	AY307935	AY307943	–	DQ017608	–	[47]
*Cookeina colensoi*	Mexico	CUP 62500	AF394040	–	–	–	–	[48]
*Cookeina colensoi*	Australia	DAR 63642	AF394038	–	–	–	–	[48]
*Cookeina colensoi*	India	FH 00432432	AF394532	–	–	–	–	[48]
*Cookeina colensoi*	New Zealand	PDD 55306	AF394037	–	–	–	–	[48]
***Cookeina cremeirosea***	**American Samoa**	**UTC000275474**	**KU306964**	–	–	–	–	[49]
*Cookeina cremeirosea*	American Samoa	UTC000275475	KU306963	–	–	–	–	[49]
***Cookeina garethjonesii*** ♦	**China**	**HKAS90509**	**KY094617**	**MG871315**	–	**MG980711**	**MG980686**	[50]
*Cookeina garethjonesii* ♦	China	HKAS90513	KY094622	MG871316	–	MG980712	MG980687	[50]
*Cookeina indica*	China	C.ind119	AF394029	–	–	–	–	[48]
*Cookeina indica* ♦	China	MFLU 16-0610	KY094621	MG871343	–	MG980727	–	[3,50]
*Cookeina indica*	Thailand	MFLU 20-0548	MT941004	–	–	–	–	[51]
*Cookeina indica *♦	China	HKAS 121171	OK170053	OK398387	OK398409	–	OK557973	This study
*Cookeina indica*	China	HKAS 121172	OK170054	–	–	–	–	This study
*Cookeina indica*	China	HKAS 121173	OK170055	–	–	–	–	This study
*Cookeina indica *♦	China	HKAS 121174	OK170058	OK398386	OK398408	–	OK557972	This study
*Cookeina insititia*	China	FH Wang sp 2	AF394033	–	–	–	–	[48]
*Cookeina insititia*	China	HMAS 70078	AF394030	–	–	–	–	[48]
*Cookeina insititia*	China	HMAS 71942	AF394031	–	–	–	–	[48]
*Cookeina korfii*	Philippines	CUP-SA-1797	KT893782	–	–	–	–	[52]
***Cookeina korfii***	**Philippines**	**CUP-SA-2454**	**KT893781**	–	–	–	–	[52]
*Cookeina sinensis*	China	HKAS 14679	AF394028	–	–	–	–	[52]
*Cookeina sinensis*	China	HMAS 70088	AF394027	–	–	–	–	[52]
*Cookeina sinensis *♦	China	HKAS 121175	OK170056	OK398385	OK398407	–	OK557971	This study
*Cookeina sinensis*	China	HKAS 121176	OK170057	–	–	–	–	This study
*Cookeina sinensis *♦	China	HKAS 121177	OK170059	OK398384	OK398406	–	OK557970	This study
*Cookeina sinensis*	China	HKAS 121178	OK170060	–	–	–	–	This study
*Cookeina sinensis*	China	HKAS 121179	OK170067	–	–	–	–	This study
*Cookeina sinensis *♦	Thailand	MFLU 21-0155	OK413269	OK398383	OK398405	–	OK557969	This study
*Cookeina speciosa*	Malaysia	C TL 6035	AF394018	–	–	–	–	[48]
*Cookeina speciosa*	Venezuela	FH Iturriaga 1C-D4	AF394011	–	–	–	–	[48]
*Cookeina speciosa*	Venezuela	FH Iturriaga 1D-D6	AF394016	–	–	–	–	[48]
*Cookeina speciosa*	Venezuela	FH Iturriaga 1E-D5	AF394003	–	–	–	–	[48]
*Cookeina speciosa*	Venezuela	FH Iturriaga 2610	AF394005	–	–	–	–	[48]
*Cookeina speciosa*	Venezuela	FH Iturriaga 2D-D4	AF394017	–	–	–	–	[48]
*Cookeina speciosa*	Venezuela	FH Iturriaga 4A-D4	AF394014	–	–	–	–	[48]
*Cookeina speciosa*	Venezuela	FH Iturriaga 7A-D4	AF394006	–	–	–	–	[48]
*Cookeina speciosa*	Colombia	FH Muneton 296	AF394013	–	–	–	–	[48]
*Cookeina speciosa*	Thailand	FH Pfister 7131	AF394009	–	–	–	–	[48]
*Cookeina speciosa*	Thailand	FH Pfister 7143	AF394010	–	–	–	–	[48]
*Cookeina speciosa *♦	Thailand	MFLU 21-0156	OK413270	OK398390	OK398412	OK585150	OK557976	This study
*Cookeina speciosa *♦	Thailand	MFLU 21-0157	OK413271	OK398391	OK398413	OK585151	OK557977	This study
*Cookeina speciosa *♦	Thailand	MFLU 21-0158	OK413272	OK398392	OK398414	OK585152	OK557978	This study
*Cookeina speciosa *♦	Thailand	MFLU 21-0159	OK413273	OK398393	OK398415	OK585153	OK557979	This study
*Cookeina speciosa*	Thailand	MFLU 21-0160	OK413274	–	–	–	–	This study
*Cookeina speciosa*	Thailand	MFLU 21-0161	OK413275	–	–	–	–	This study
*Cookeina speciosa*	Thailand	MFLU 21-0162	OK413276	–	–	–	–	This study
*Cookeina speciosa*	China	HKAS 121180	OK170044	–	–	–	–	This study
*Cookeina speciosa*	China	HKAS 121181	OK170045	–	–	–	–	This study
*Cookeina speciosa*	China	HKAS 121182	OK170047	–	–	–	–	This study
*Cookeina speciosa*	China	HKAS 121183	OK170048	–	–	–	–	This study
*Cookeina speciosa*	China	HKAS 121184	OK170049	–	–	–	–	This study
*Cookeina speciosa*	China	HKAS 121185	OK170050	–	–	–	–	This study
*Cookeina speciosa*	China	HKAS 121186	OK170064	–	–	–	–	This study
*Cookeina speciosa*	China	HKAS 121187	OK170065	–	–	–	–	This study
*Cookeina speciosa*	China	HKAS 121188	OK170066	–	–	–	–	This study
*Cookeina speciosa*	China	HKAS 124640	OP364889	–	–	–	–	This study
*Cookeina sulcipes*	Thailand	MFLU 15-2358	KY094620	–	–	–	–	[50]
*Cookeina tricholoma*	Thailand	FH Pfister 7170	AF394020	–	–	–	–	[48]
*Cookeina tricholoma* ♦	China	HKAS87041	KY094619	MG871317	–	–	MG980688	[3,50]
*Cookeina tricholoma* ♦	Thailand	MFLU 15-2359	KY094618	MG871318	MG859240	–	MG980689	[3,50]
*Cookeina tricholoma *♦	Thailand	MFLU 21-0165	OK413279	OK398394	OK398416	–	–	This study
*Cookeina tricholoma *♦	Thailand	MFLU 21-0166	OK413280	OK398395	OK398417	–	OK557980	This study
*Cookeina tricholoma *♦	Thailand	MFLU 21-0167	OK413281	OK398396	OK398418	–	OK557981	This study
*Cookeina tricholoma*	Thailand	MFLU 21-0168	OK413282	OK398397	OK398419	–	–	This study
*Cookeina tricholoma*	Thailand	MFLU 21-0169	OK413283	OK398398	OK398420	–	–	This study
*Cookeina tricholoma*	Thailand	MFLU 21-0163	OK413277	–	–	–	–	This study
*Cookeina tricholoma*	Thailand	MFLU 21-0164	OK413278	–	–	–	–	This study
*Cookeina tricholoma*	China	HKAS 121189	OK170043	–	–	–	–	This study
*Cookeina tricholoma*	China	HKAS 121190	OK170046	–	–	–	–	This study
*Cookeina tricholoma*	China	HKAS 121191	OK170061	–	–	–	–	This study
*Cookeina venezuelae*	Puerto Rico	FH00432502	AF394041	–	–	–	–	[48]
*Cookeina venezuelae*	Venezuela	FH Iturriaga 6065	AF394044	–	–	–	–	[48]
*Cookeina venezuelae*	Venezuela	FH Iturriaga 6066	AF394043	–	–	–	–	[48]
*Cookeina venezuelae*	Guadeloupe	FH00432503	AF394042	–	–	–	–	[48]
*Geodina guanacastensis* ♦	Bahamas	FH	MN096939	MN096940	MN096941	MN103424	MN090946	[23]
***Geodina guanacastensis***	**Costa Rica**	**CUP CA84**	**MN096938**	–	–	–	–	[23]
*Geodina guanacastensis*	Dominican Republic	JBSD 127408	MG597289	–	–	–	–	[22,23]
*Geodina guanacastensis*	Dominican Republic	JBSD 127409	MG597290	–	–	–	–	[22,23]
*Kompsoscypha chudei* ♦	China	HKAS 107663	MT907443	MT907444	–	–	–	[51]
*Kompsoscypha phyllogena* ♦	Puerto Rico	DHP 10-690	–	JQ260810	JQ260820	MN103430	–	[15]
*Microstoma floccosum*	Mexico	FH K. Griffith (Micro45)	AF394046	–	–	–	–	[48]
*Microstoma floccosum*	Mexico	FH K. Griffith (Micro46)	AF394045	–	–	–	–	[48]
*Nanoscypha striatispora*	China	HMAS 61133	U66016	–	–	–	–	[21]
*Nanoscypha tetraspora* ♦	Puerto Rico	FH 00464570	AF117352	DQ220374	AF006314	–	–	[21,53,54]
***Nanoscypha aequispora ***♦	**Thailand**	**MFLU 21-0170**	**OK413284**	**OK398399**	**OK398421**	**OK585154**	**–**	**This study**
*Nanoscypha aequispora *♦	Thailand	MFLU 21-0171	OK413285	OK398400	OK398422	OK585155	OK557982	This study
*Neournula pouchetii*	USA	MO 205345	KT968605	–	–	–	–	[55]
*Phillipsia carnicolor* ♦	Thailand	DHP-7126 (FH)	AF117353	JQ260811	JQ260821	MN103426	MN090948	[53]
*Phillipsia carnicolor*	Thailand	MFLU 18-0713	MH602282	–	–	–	–	[56]
***Phillipsia chinensis***	**China**	**HMAS 76094**	**AY254710**	**–**	**–**	**–**	**–**	[57]
*Phillipsia crispata*	Ecuador	T. Læssøe AAU-44801	AF117354	–	–	–	–	[53]
*Phillipsia crispata* ♦	Ecuador	T. Læssøe AAU-44895a	AF117355	AY945845	–	DQ017599	–	[47]
*Phillipsia domingensis*	USA	CO-1864 (NO)	AF117363	–	–	–	–	[53]
*Phillipsia domingensis*	Costa Rica	CO-2032 (NO)	AF117361	–	–	–	–	[53]
*Phillipsia domingensis* ♦	Thailand	DHP 7169 (FH)	AF117373	JQ260817	JQ260827	–	–	[53]
*Phillipsia domingensis*	Dominican Republic	DR-321 (CFMR)	AF117370	–	–	–	–	[53]
*Phillipsia domingensis*	Costa Rica	Franco-M 1270 (NY)	AF117358	–	–	–	–	[53]
*Phillipsia domingensis*	Puerto Rico	PR-1583 (FH)	AF117365	–	–	–	–	[47]
*Phillipsia domingensis *♦	China	HKAS 121192	OK170062	OK398388	OK398410	OK585148	OK557974	This study
*Phillipsia domingensis *♦	China	HKAS 121193	OK170063	OK398389	OK398411	OK585149	OK557975	This study
***Phillipsia gelatinosa*** ♦	**Thailand**	**MFLU 15-2360**	**KY498595**	**KY498589**	**–**	**MG980728**	**–**	[58]
***Phillipsia gelatinosa***	**Thailand**	**MFLU 16-2956**	**KY498593**	**–**	**–**	**–**	**–**	[58]
***Phillipsia hydei***	**Thailand**	**MFLU 18-0714**	**MH602283**	**–**	**–**	**–**	**–**	[56]
*Phillipsia hydei*	Thailand	MFLU 18-1329	MH602284	–	–	–	–	[56]
*Phillipsia lutea* ♦	French Guiana	NY-4113 (NY)	AF117374	JQ260816	JQ260826			[53]
*Phillipsia olivacea*	Costa Rica	Franco-M 1360 (NY)	AF117375	–	–	–	–	[53]
*Phillipsia olivacea* ♦	Venezuela	Halling-5456 (NY)	AF117376	JQ260814	JQ260824	–	–	[53]
*Phillipsia olivacea*	Ecuador	T. Læssøe AAU-43162 (C)	AF117378	–	–	–	–	[47]
*Phillipsia subpurpurea*	China	MFLU 16-0612	KY498596	–	–	–	–	[58]
*Pithya cupressina* ♦	USA	mh 208	U66009	JQ260818	AF006316	–	–	[23,59]
*Pithya sp.*	China	DWS8m3	KJ188703					[60]
*Pithya sp.*	USA	T5N32c	AY465469	–	–	–	–	[61]
*Pithya vulgaris*	–	RK 90.01	U66008	–	–	–	–	[59]
***Pithya villosa ***♦	**China**	**HKAS 104653**	**OK170069**	**OK398401**	**OK398423**	**OK585156**	**–**	**This study**
*Pithya villosa *♦	China	HKAS 121194	OK170068	OK398402	OK398424	–	–	This study
*Plectania nannfeldtii* ♦	USA	FH 00822732	–	AY945853	–	DQ017592	KC109214	[47,62]
*Pseudopithyella minuscula* ♦	USA	FH 00465568	–	AY945849	AF006317	DQ017600	FJ238387	[47]
*Rickiella edulis* ♦	Argentina	BAFC 51697	JQ260808	JQ260809	JQ260819	MN103425	MN090947	[15]
*Sarcoscypha austriaca*	Norway	CUP 62771	U66010	–	–	–	–	[59]
*Sarcoscypha austriaca*	USA	CUP 63162	U66011	–	–	–	–	[59]
*Sarcoscypha coccinea* ♦	–	AFTOL-ID 50	DQ491486	AY544647	–	DQ497612	–	[63]
*Sarcoscypha coccinea* ♦	France	AFTOL-ID 930	–	FJ176859	FJ176805	FJ713615	–	[63]
*Sarcoscypha coccinea*	USA	CUP 62113	U66013	–	–	–	–	[59]
*Sarcoscypha coccinea*	USA	CUP 63160	U66015	–	–	–	–	[59]
*Sarcoscypha dudleyi*	USA	CUP 62775	U66018	–	–	–	–	[59]
*Sarcoscypha dudleyi*	China	HMJAU36044	KU234218	–	–	–	–	[64]
*Sarcoscypha dudleyi*	–	mh 192	U66019	–	–	–	–	[59]
*Sarcoscypha emarginata*	Luxembourg	CUP 62723	U66020	–	–	–	–	[59]
*Sarcoscypha emarginata*	–	HB2861	U66021	–	–	–	–	[59]
*Sarcoscypha hosoyae*	–	TRL 456	U66031	–	–	–	–	[59]
*Sarcoscypha humberiana*	China	TNM F28630	KT716833	–	–	–	–	[65]
*Sarcoscypha humberiana*	China	CUP 63489	U66028	–	–	–	–	[59]
*Sarcoscypha javensis*	China	HMAS 61198	U66026	–	–	–	–	[59]
*Sarcoscypha knixoniana*	–	TRL 1006	U66030	–	–	–	–	[59]
*Sarcoscypha korfiana*	–	mh 705	AF026308	–	–	–	–	[21]
***Sarcoscypha longitudinalis ***♦	**China**	**HKAS 121195**	**OK170051**	**OK398403**	**OK398425**	**OK585157**	**–**	**This study**
*Sarcoscypha longitudinalis *♦	China	HKAS 121196	OK170052	OK398404	OK398426	–	–	This study
*Sarcoscypha macaronesica*	Canary Islands	CUP-MM 2628	U66022	–	–	–	–	[59]
*Sarcoscypha macaronesica*	–	TFC-MIC 6460	U66023	–	–	–	–	[59]
*Sarcpscypha mesocyatha*	China	TNM F3688	KT936558	–	–	–	–	[65]
*Sarcpscypha mesocyatha*	China	TNM F5134	KT936559	–	–	–	–	[65]
*Sarcoscypha mesocyatha*	USA	CUP 62699	U66029	–	–	–	–	[59]
***Sarcoscypha minuta***	**China**	**TNM F28831**	**KT716834**	**–**	**–**	**–**	**–**	[65]
*Sarcoscypha occidentalis*	USA	CUP 62777	U66024	–	–	–	–	[59]
*Sarcoscypha occidentalis*	USA	CUP 63484	U66025	–	–	–	–	[59]
*Sarcoscypha sp.*	China	HMAS 61202	U66027	–	–	–	–	[59]
*Sarcoscypha tatakensis*	China	TNM F0754	KT716835	–	–	–	–	[65]
***Sarcoscypha tatakensis***	**China**	**TNM F0993**	**KT716836**	**–**	**–**	**–**	**–**	[65]
*Sarcoscypha vassiljevae* ♦	Chian	HKAS 89817	MG871302	MG871337	–	MG980724	MG980700	[3]
*Sarcoscypha vassiljevae*	China	HMAS 61210	U66017	–	–	–	–	[59]
*Urnula craterium* ♦	USA	DHP 04-511	–	AY945851	–	DQ017595	KC109216	[47,62]
*Wynnea americana* ♦	USA	FH 00445979	MK599141	AY945848	MK592785	MN103435	MN103417	[23,66]
*Wynnea americana*	USA	HKAS 75484	MG871308	–	–	–	–	[3]
*Wynnea gigantea*	China	HKAS 101385	MG871307	–	–	–	–	[3]
*Wynnea macrospora* ♦	China	FH 00445975	MK335784	MK335803	MK335793	MN103432	MN103419	[23,66]
*Wynnea macrospora* ♦	–	CUP 2684	–	MK335804	MK335795	–	MN103420	[23,66]
*Wynnea sparassoides* ♦	USA	FH 00445986	–	EU360917	MK335796	MN103431	MN103418	[23,47]

^1^ Names in red indicate newly-described species in this study. Names in bold indicate type collections. Names in blue indicate newly-sequenced collections. ^2^ Species names marked by “♦” refer to taxa used in the combined tree. ^3^ Abbreviations: AAU: Herbarium of Aarhus University, Denmark; BAFC: Facultad de Ciencias Exactas y Naturales, Argentina; CFMR: Center for Forest Mycology Research, USDA Forest Service, USA; CUP: The Cornell Plant Pathology Herbarium, New York, USA; DAR: NSW Plant Pathology & Mycology Herbarium, Australia; FH: Farlow Herbarium, Harvard University Herbaria, Cambridge, Massachusetts, USA; HKAS: Herbarium of Cryptogams of Kunming Institute of Botany, Chinese Academy of Sciences, China; HMAS: Herbarium Mycologicum Academiae Sinicae, Beijing, China; HMJAU: Herbarium of Mycology of Jilin Agricultural University, Changchun, China; JBSD: Herbarium of the Santo Domingo National Botanical Garden, Dominican Republic; MFLU: Mae Fah Luang University Herbarium, Chiang Rai, Thailand; MO: Missouri Botanical Garden Herbarium, Missouri, USA; NY: New York Botanical Garden, USA; PDD: New Zealand Fungarium, New Zealand; TFC: La Laguna University Herbarium, Spain; TNM: Department of Botany, National Museum of Natural Science, Taiwan, China; UTC: Intermountain Herbarium, Utah, USA.

## 3. Results

### 3.1. Phylogenetic Analysis

**Figure 1:**
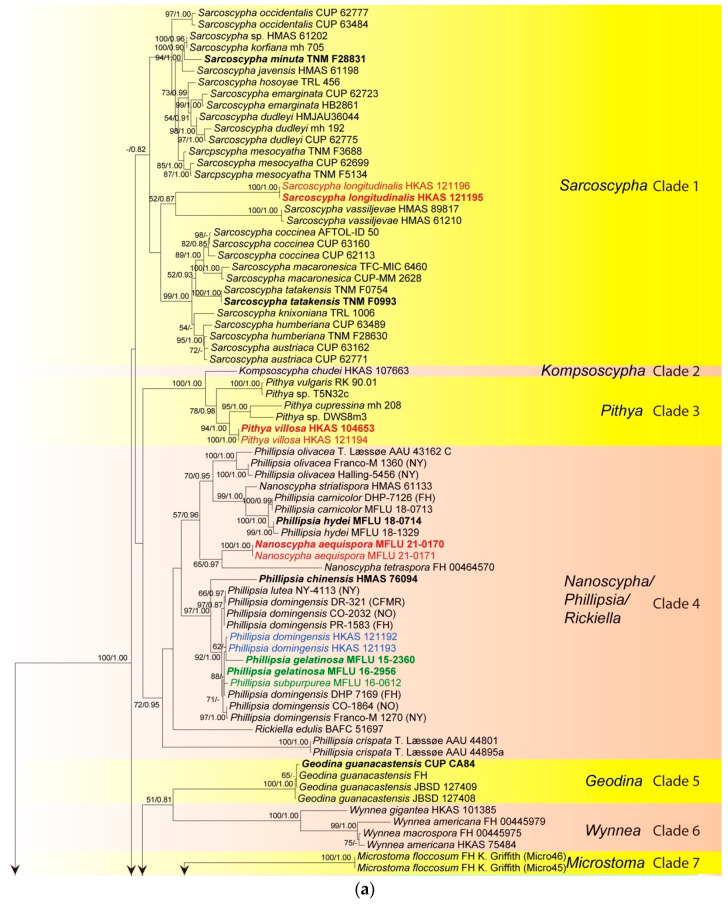

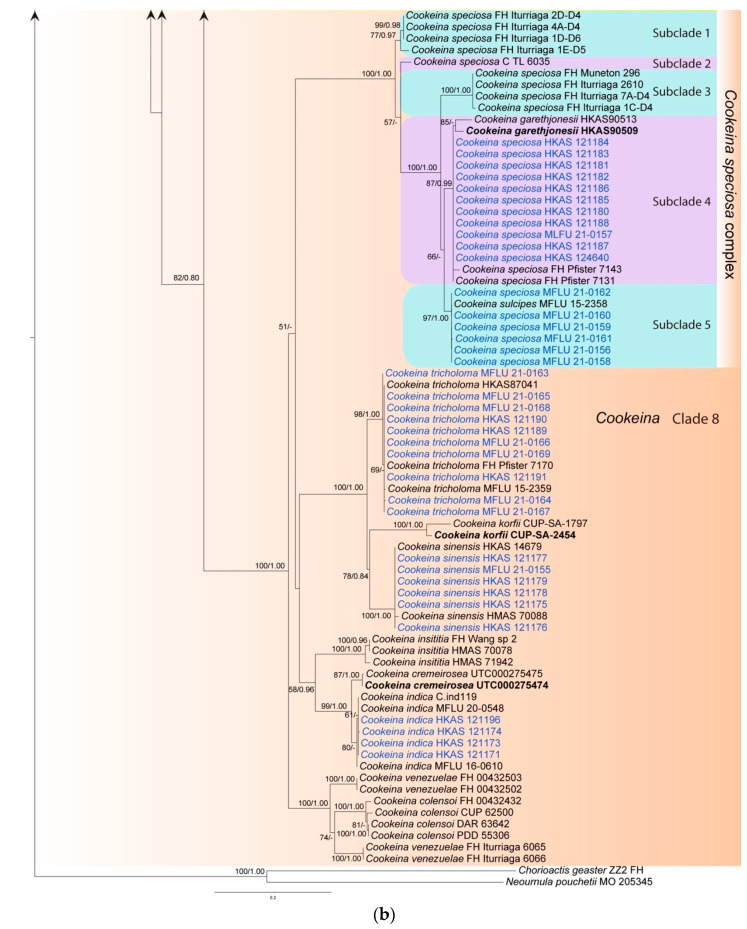
Maximum likelihood tree of ITS sequence data inferred from 151 taxa and 476 sites under the GTR (general time reversible) + G + I model of nucleotide substitution. Bootstrap support values for maximum likelihood (BS) and Bayesian posterior probabilities (PP) greater than 50% and 0.80 are indicated above or below the nodes in this order. Names in red indicate newly-described species and names in blue stand for newly-sequenced collections. Names in green indicate correction to *Phillipsia domingensis* (**a**). *Chorioactis geaster* (ZZ2 FH) and *Neournula pouchetii* (MO 205345) are used as the outgroup taxa (**b**).

The ITS phylogenetic tree was inferred (Figure 1) using 151 taxa and 476 sites including sequences from *Chorioactis geaster* (Peck) Kupfer (ZZ2 FH) and *Neournula pouchetii* (Berthet & Riousset) Paden (MO 205345) as outgroup. The best sorting RAxML tree had a final likelihood value of −9665.136236. Sequences of the ITS region are available for nearly all taxa of *Sarcoscyphaceae* for which molecular data exist. Genera are grouped in distinct monophyletic clades except for *Phillipsia*, *Nanoscypha* and *Rickiella*, which are paraphyletic. In the ITS tree, taxa are grouped in eight main clades, which mainly represent genera. Our new collections were placed in four clades, namely clade 1, clade 3, clade 4, and clade 8. Within clade 1 (*Sarcoscypha* clade), the newly-described *Sarcoscypha* species, *S. longitudinalis* (represented by two collections), formed an individual branch as sister to *S. vassiljevae* Raitv., but this relationship was not strongly supported (52BS/0.87PP). Clade 2 comprised a single species, *K. chudei* (Pat. ex Le Gal) Pfister, and was sister to clade 3 (100BS/1.00PP). Within clade 3 (*Pithya* clade), our new *Pithya* species (represented by two collections) branched sister to *Pi. cupressina* (Batsch) Fuckel (mh 208) and to one unknown *Pithya* species (DWS8m3) with strong statistical support (94BS/1.00PP). Clade 4 comprised *Phillipsia*, *Rickiella*, and *Nanoscypha*. The new species, *N. aequispora*, was sister to *N. tetraspora* (Seaver) Denison (DHP PR-61) with moderate support (65BS/0.97PP). Two new collections of *Ph. domingensis* (HKAS 121192 and HKAS 121193) were placed within the *Ph. domingensis* complex. Clades 5, 6, and 7 comprised *Geodina* (one species), *Wynnea* (four species) and *Microstoma* (one species). Clade 8 (*Cookeina* clade) contained the rest of the new collections. Seventeen new collections were distributed in two of the five subclades within the *C. speciosa* complex, ten grouped with *C. tricholoma*, six clustered with *C. sinensis* Zheng Wang, while four grouped with *C. indica* Pfister & R. Kaushal.

**Figure 2:**
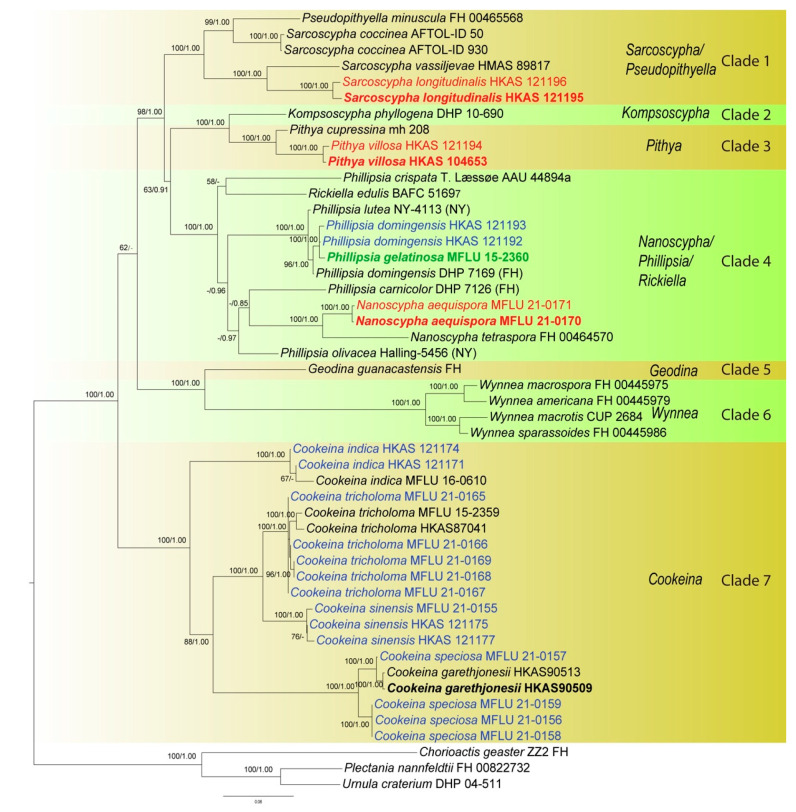
Phylogenetic tree of combined ITS, LSU, SSU, *rpb2*, and *tef-1α* sequence data inferred from 49 taxa and 4366 sites under the GTR + G + I model of nucleotide substitution. Numerical values at the nodes indicate maximum likelihood bootstrap support (BS) and posterior probabilities (PP). Values of BS greater than 50% and PP over 0.80 are indicated above or below the nodes in this order. Names in red indicate newly-described species and names in blue stand for newly-sequenced collections. Names in green indicate correction to *Phillipsia domingensis*. Tree is artificially rooted to *Chorioactis geaster* (ZZ2 FH), *Plectania nannfeldtii* (FH 00822732) and *Urnula criterium* (DHP 04-511).

Initially, a phylogenetic tree was inferred using a combined dataset of ITS, LSU, SSU, *rpb2*, and *tef-1α* data containing all available strains in the preliminary analysis (data not shown). However, the large amount of missing data (for many strains only ITS was available, while for others only LSU) confounded the results as indicated by unstable placement of taxa and very low statistical support in deep and shallow nodes. Therefore, a smaller representative dataset was assembled containing 49 taxa, for which a minimum of three genes was available for each taxon (Figure 2). The alignment comprised 4366 total characters (ITS: 1–482 bp; LSU: 483–1386 bp; SSU: 1387–2432 bp; *rpb2*: 2433–3485 bp; *tef-1α*: 3486–4366 bp). The best-sorting RAxML tree had a final likelihood value of −30228.032462. In the combined data tree, taxa grouped in seven main clades. The *Microstoma* clade (clade 7 in the ITS tree) is missing due to lack of data. *Kompsoscypha*, *Pithya*, *Wynnea*, *Geodina* and *Cookeina* were monophyletic. Species of *Phillipsia*, *Nanoscypha* and *Rickiella* interspersed within a clade, while *Pseudopithyella* grouped within *Sarcoscypha*. The phylogenetic placements of our new species and collections were almost identical to that of the ITS tree, but statistical supports were much higher in the combined data tree (Figure 1 and Figure 2). *Pseudopithyella* did not separate from *Sarcoscypha* but grouped as sister to *S. coccinea* in the combined genes tree with nearly maximum statistical support (99BS/1.00PP). It is unknown if this relationship is recovered in the ITS tree as the sequence is not available. However, the relationship was recovered in the single gene trees that contain sufficient taxon sampling for *Sarcoscypha*, i.e., *rpb2* and SSU.

### 3.2. Taxonomy

**Figure 3:**
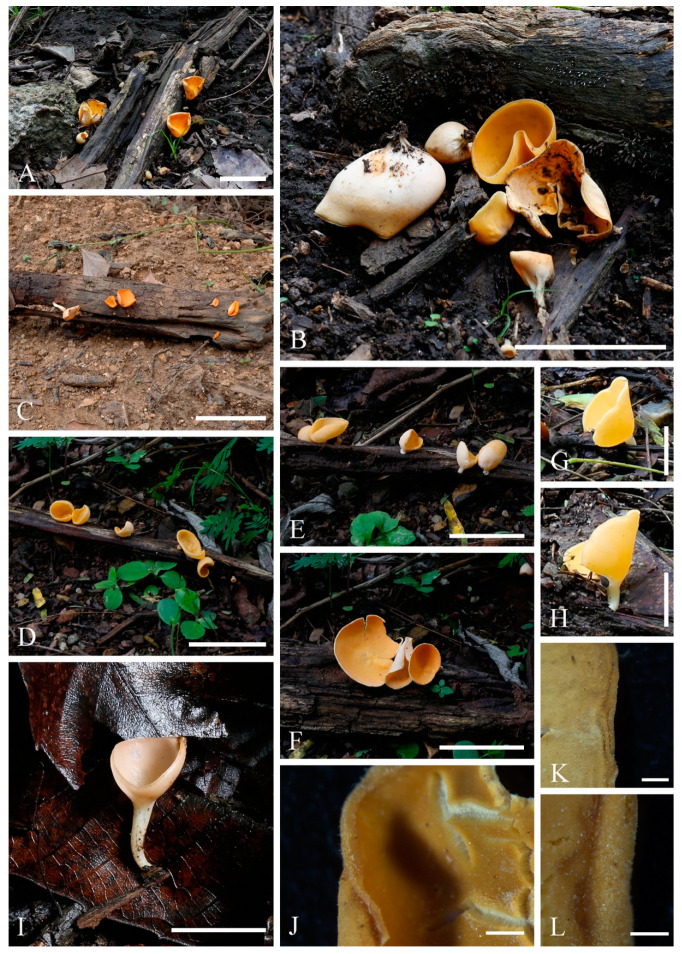
***Cookeina indica***. (**A**–**I**) Fresh apothecia [(**A**,**B**) HKAS 121171. (**C**) HKAS 121172. (**D**–**H**) HKAS 121173. (**I**) HKAS 121174.] (**J**) Margin (HKAS 121173). (**K**) Receptacle surface of an apothecium (HKAS 121173). (**L**) A concentric sulcus (HKAS 121173). Scale bars (**A**,**B**,**F**) = 5 cm; (**C**–**E**) = 10 cm; (**G**–**I**) = 3 cm; (**J**,**K**) = 1000 μm; (**L**) = 500 μm.

**Figure 4:**
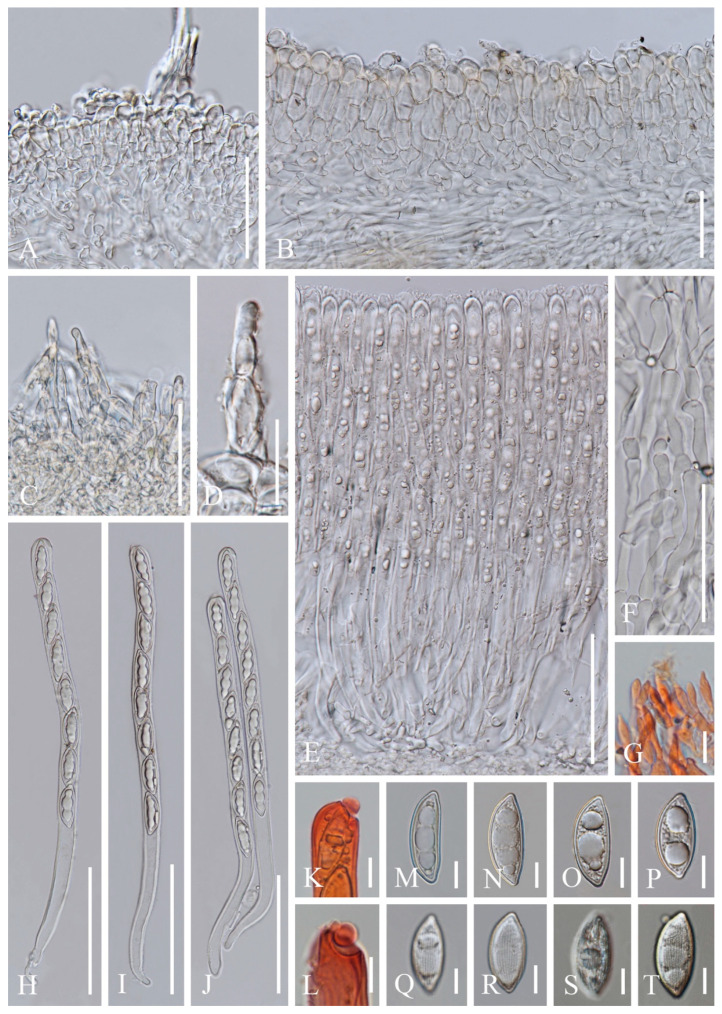
***Cookeina indica*** (HKAS 121173). (**A**) Vertical section of stipe ectal excipulum. (**B**) Vertical section of receptacle ectal excipulum. (**C**) Hyphoid hairs at the margin. (**D**) A moniliform hair-like processes. (**E**) Hymenium. (**F**) Paraphyses. (**G**) Apical part of the paraphyses in CR. (**H**–**J**) Asci and ascospores. (**K**,**L**) Apices of asci in CR. (**M**–**T**) Ascospores ornamented with longitudinal striate ridges. Scale bars (**A**–**C**,**F**) = 50 μm; (**D**) = 20 μm; (**E**,**H**–**J**) = 100 μm; (**G**,**K**–**T**) = 10 μm.

1. ***Cookeina indica*** Pfister & R. Kaushal, Mycotaxon 20(1): 117 (1984); Figure 3 and Figure 4

Index Fungorum number: IF 302844; Facesoffungi number: FoF 02671

*Saprobic* on dead wood. **Teleomorph**: *Apothecia* up to 7 cm high, 1–4 cm broad, solitary or scattered, deeply cupulate, rarely ear-shaped, fleshy, with short to long stipe. *Stipe* up to 4 cm long, up to 3 mm broad, central to eccentrical, terete, or sharply reduced to a basal, sulcate attachment, solid, usually white to yellowish when fresh, yellow when dry, nearly smooth. *Receptacle* cup-shaped, surface light ivory (RAL 1015), yellowish to orange when fresh, nearly smooth, with one concentric sulcus, margin broadly entire, or rarely deeply split on one side. *Disc* deeply concave, mostly concolorous with the receptacle surface, or somewhat darker in colour. *Stipal ecto-excipulum* 70–140 µm broad, composed of hyaline to yellowish *textura globulosa-angularis*, cells 14–19 × 11–14 μm, some outermost globose cells form irregularly loose aggregates to a pruinose-like surface, rarely with hyphoid hairs which are 3–6 µm wide, hyaline, septate, slightly tapering towards a rounded apex, usually fasciculate. *Stipal medulla* composed of hyaline to subhyaline *textura intricata*, hyphae 3–6.5 μm broad. *Ectal excipulum* 80–130 µm thick, mainly divided to two sub-layers delimited by outer and inner cells: outer layer composed of hyaline to yellowish *textura globulosa* to *textura prismatica*, terminal cells globose, 13–19 μm diameter, with a pruinose-like surface, prismatic cells 23–31 × 11–16 μm, hyphoid hairs forming abundant fascicles at margin, composed of 4–6 µm wide subhyaline, septate hyphae, sometimes with individual monilioid hairlike processes having 2–3 ellipsoid cells; inner layer composed of hyaline *textura angularis* to *textura epidermoidea*, angular cells 14–18 × 11–14 μm, and elongated cells 16–23 × 6–9 μm. *Medullary excipulum* 200–260 μm broad, composed of hyaline *textura intricata*, hyphae 4–7 μm broad. *Hymenium* 340–400 μm thick, subhyaline, paraphyses exceeding the asci slightly when dehydrated. *Paraphyses* 4–6 μm broad in the middle part, cylindric, septate, constricted at septa, normally anastomosing to form a network, branched in the apical part, apical cell tapered. *Asci* 330–360 × 15–19 μm, 8-spored, eccentrically operculate, cylindrical, with obtuse apices and attenuated basal part. *Ascospores* [20/1/1, in H_2_O] (26.3–)29.8–36(–38) × (10.8–)11.5–13.4(–14.2) μm (Q = 2.08–3.13, **Q** = 2.64 ± 0.28), fusiform to subreniform, inequilateral, subpapillate at the poles, uniseriate, often biguttulate to 3-guttulate, some multiguttulate, ornamented with parallel ridges arranged longitudinally. **Anamorph**: not seen.

Material examined: CHINA, YUNNAN, XISHUANGBANNA: Menghai, on unidentified dead branch and trunk under broadleaved forest, 19 August 2019, Ming Zeng, ZM255 (HKAS 121171), ZM257 (HKAS 121172); Mengyang, on unidentified dead branch under broadleaved forest, 21 August 2019, Ming Zeng, ZM280 (HKAS 121173); Jinghong, on unidentified dead twigs under broadleaved forest, 23 August 2019, Ming Zeng, ZM306 (HKAS 121174).

Notes: This species is distinguished by nearly smooth apothecia with a concentric sulcus close to margin, paraphyses with tapering ends, fusiform and inequilateral ascospores with subpapillate ends, and longitudinal striae on surface of ascospores [67]. *Cookeina indica* was first discovered in India and has since then been reported in southwestern China [6,50,67,68,69]. It was also recently discovered in Thailand [51]. This species has a nearly smooth surface when observed with the naked eye, compared to most other species that have easily visible hairs [67], while the furfuraceous receptacle surface can be seen with a magnifying hand lens. *Cookeina cremeirosea* Kropp has smooth apothecia, and it showed a close phylogenetic relationship with *C. indica* (Figure 1) [49]. *Cookeina cremeirosea* is distinct in having pinkish apothecia and smooth-walled ascospores [49]. Compared with the type description in the protologue [68], our collections have large apothecia (up to 7 cm high vs up to 3.5 cm high) and a long stipe (up to 4 cm long vs up to 2.2 cm long). In addition, ascospores (11.5–13.4 μm vs 10–11.5 μm) also vary significantly in width.

**Figure 5:**
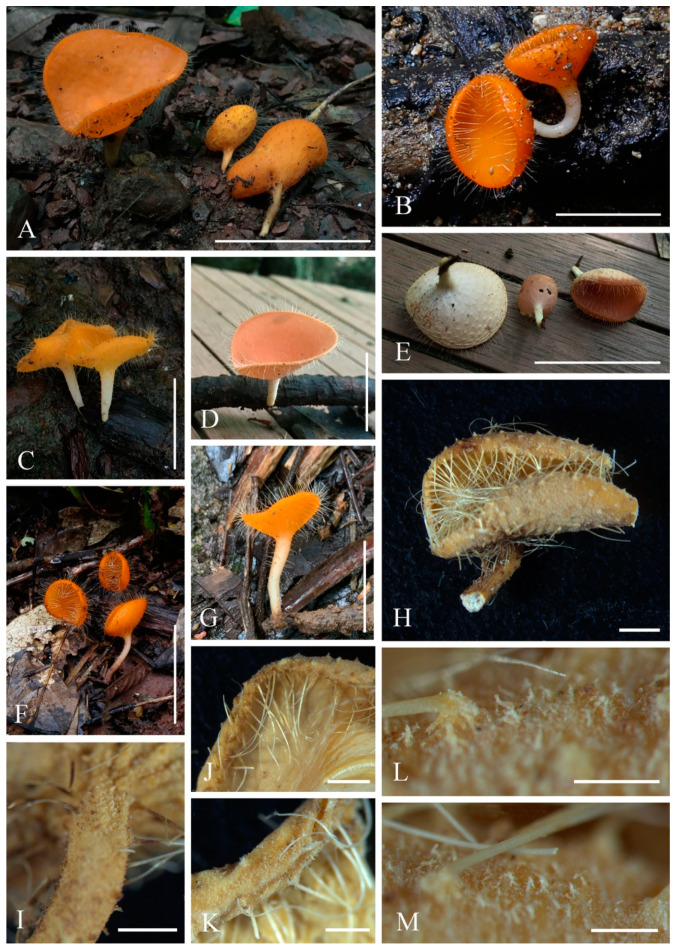
***Cookeina sinensis***. (**A**–**G**) Fresh apothecia [(**A**) HKAS 121177. (**B**) HKAS 121176. (**C**) HKAS 121178. (**D**,**E**) HKAS 121179. (**F**) HKAS 121175. (**G**) MFLU 21-0155.] (**H**) Dry apothecium (HKAS 121175). (**I**) Stipe (HKAS 121175). (**J**) Compound hairs (HKAS 121177). (**K**) Margin (HKAS 121178). (**L**,**M**) Receptacle surface of an apothecium (HKAS 121175). Scale bars (**A**,**E**,**F**) = 5 cm; (**B**–**D**,**G**) = 3 cm; (**H**,**J**) = 2000 μm; (**I**,**K**) = 1000 μm; (**L**,**M**) = 500 μm.

**Figure 6:**
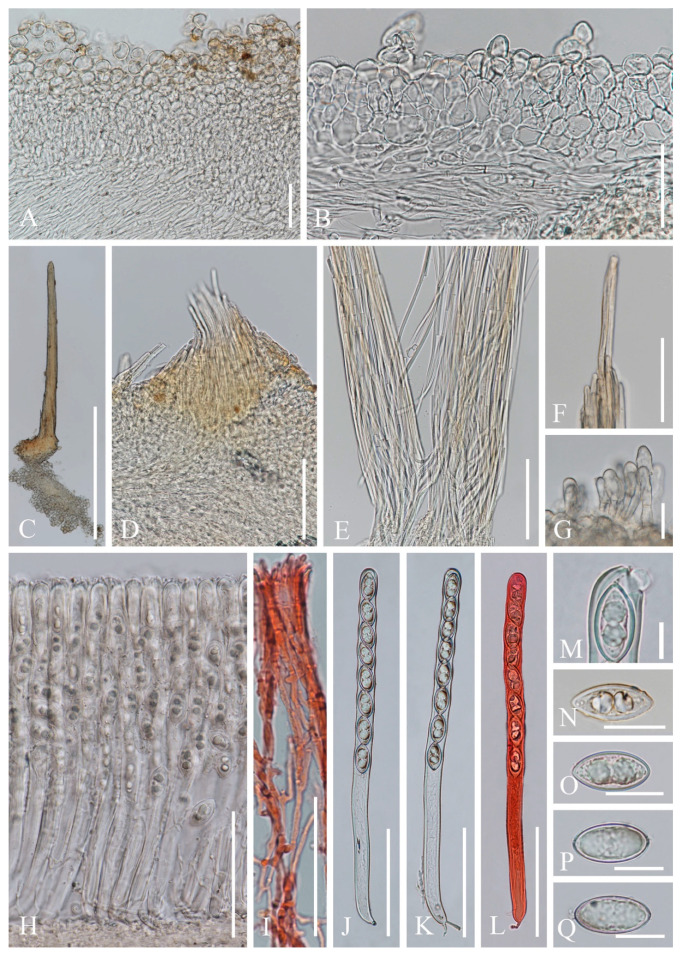
***Cookeina sinensis*** (HKAS 121179). (**A**) Vertical section of stipe ectal excipulum. (**B**) Vertical section of receptacle ectal excipulum. (**C**) Compound hair. (**D**) Base of the compound hair arising from medullary excipulum. (**E**) Loose compound hairs. (**F**) Tips of compound hairs. (**G**) Hyphoid hairs. (**H**) Hymenium. (**I**) Paraphyses in CR. (**J**–**L**) Asci and ascospores (**L**) Ascus and ascospores in CR. (**M**) Apex of an ascus. (**N**–**Q**) Ascospores. Scale bars (**A**,**B**,**F**,**I**) = 50 μm; (**C**) = 500 μm; (**D**,**E**,**H**,**J**–**L**) = 100 μm; (**G**,**N**–**Q**) = 20 μm; (**M**) = 10 μm.

2. ***Cookeina sinensis*** Zheng Wang, Mycotaxon 62: 293 (1997); Figure 5 and Figure 6

Index Fungorum number: IF 437499; Facesoffungi number: FoF 02674

*Saprobic* on dead wood. **Teleomorph**: *Apothecia* up to 4 cm high, up to 5 cm broad, scattered, cupulate, fleshy, with conspicuous stipe. *Stipe* up to 2 cm long, up to 8 mm broad, central, terete, solid, white to yellowish when fresh, yellow when dry, furfuraceous or tomentose, with long compound hairs as on the receptacle. *Receptacle* cup-shaped, surface yellowish to orange, or rarely oyster white (RAL 1013) when fresh, with long compound hairs, tomentose, margin entire, leveled or inrolled. *Disc* deeply concave, yellow to orange, or pinkish when fresh, mostly concolorous with the receptacle surface. *Stipal ecto-excipulum* 90–260 µm broad, composed of hyaline to yellowish *textura globulosa-angularis*, cells 16–19 × 12–15 μm, some outer globose cells irregularly loosely aggregated forming a pruinose-like surface. *Stipal medulla* composed of hyaline to subhyaline *textura intricata*, hyphae 4–6.5 μm broad. *Ectal excipulum* 50–110 µm thick, composed of hyaline *textura globulosa-angularis*, cells 15–20 × 12–15 μm, with hyphoid hairs forming abundant fascicles, composed of 4–7 µm wide, subhyaline, septate, broad hyphae, sometimes with monilioid processes having 1–2 globose cells forming a pruinose-like surface. *Medullary excipulum* 140–220 μm thick, composed of hyaline *textura intricata*, hyphae interwoven, 3–6 μm wide. *Compound hairs* up to 5 mm long, up to 150 µm diameter at base of fascicle, fasciculate, yellow to brown, arising from the medullary excipulum, composed of parallel, yellowish, septate, thick-walled individial hairs, 4–5 µm diameter, with a rounded end. *Hymenium* 280–310 μm thick, subhyaline. *Paraphyses* 3–4 μm broad at the middle, filiform, septate, anastomosing to form a network in middle parts, branched, with a rounded end. *Asci* 290–300 × 15–18 μm, 8-spored, eccentrically operculate, cylindrical, with obtuse apices and slightly attenuated basal part. *Ascospores* [20/1/1, in H_2_O] (25–)26.3–29.6(–30.4) × (12.2–)12.4–13.7(–14.2) μm (Q = 1.91–2.38, **Q** = 2.15 ± 0.14), fusiform, pointed at ends, uniseriate, equilateral, biguttulate, rarely apiculi-like solidifications present at one pole, smooth. **Anamorph**: not seen.

Material examined: CHINA, YUNNAN, XISHUANGBANNA: Dadugang, on unidentified dead branch under broadleaved forest, 22 August 2019, Ming Zeng, ZM287 (HKAS 121175); Jinghong, on unidentified dead branch under broadleaved forest, 23 August 2019, Ming Zeng, ZM297 (HKAS 121176); Manshan, on unidentified dead branch under broadleaved forest, 25 August 2019, Ming Zeng, ZM320 (HKAS 121177), ZM322 (HKAS 121178); Mengla, on unidentified dead branch under broadleaved forest, 27 August 2019, Ming Zeng, ZM351 (HKAS 121179); THAILAND: Chiangmai, Mushroom Research Center (MRC), on unidentified dead branch under broadleaved forest, 16 August 2020, Deping Wei, ZM370 (MFLU 21-0155).

Notes: This species has long compound hairs covering receptacle, arising from medullary excipulum. The ascospores are fusiform, smooth, with apiculi-like structure [6,67]. *Cookeina sinensis* is similar to *C. tricholoma* and *C. korfii* Iturr., F. Xu & Pfister, as all three share the conspicuous long compound hairs. Compared with *C. korfii* and *C. sinensis*, *C. korfii* has smaller ascospores (22–25 × 9–11.5 μm) [52]. The major difference between *C. sinensis* and *C. tricholoma* is that the ascospores of *C. tricholoma* have longitudinal striae, whereas *C. sinensis* has smooth ascospores [67]. *Cookeina sinensis* had only been reported from China [6,67,70], until Patil et al. [71] described new collections from India. Comparing Indian specimens with ours, the Indian specimens have larger ascospores (30–40 × 15.6 μm) [71]. The ascospores of our specimens have similar size with type specimens (25–28 × 12–12.5 μm) described by Wang [70], which have smaller apothecia (2.5 cm high) than ours. In this study, we report on a new record collected from Thailand (MFLU 21-0155).

**Figure 7:**
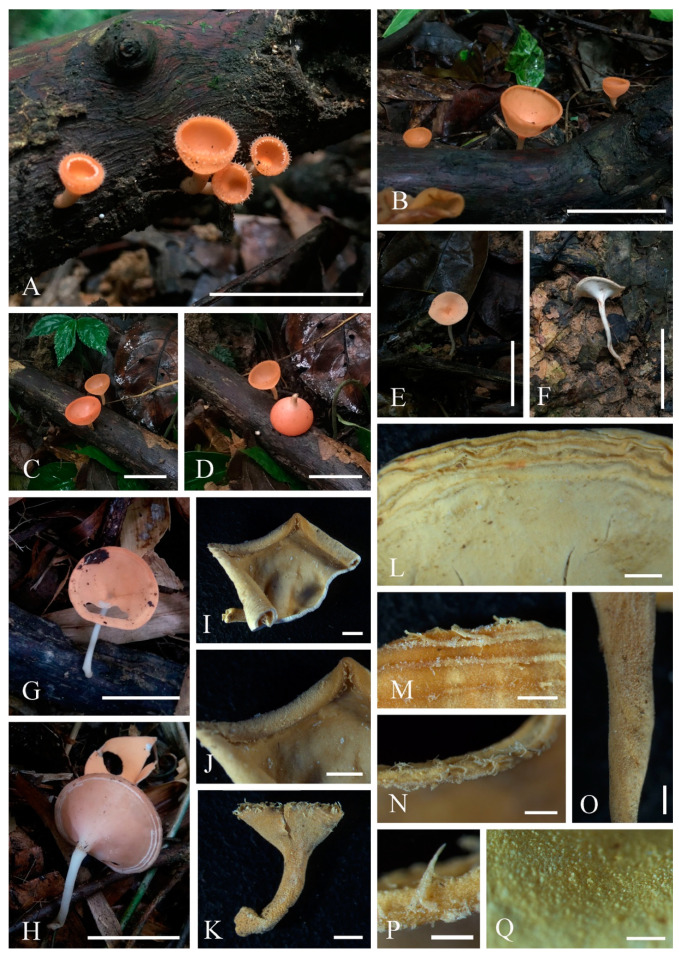
***Cookeina speciosa***. (**A**–**H**) Fresh apothecia [(**A**,**B**). HKAS 121188. (**C**,**D**) HKAS 121186. (**E**,**F**) HKAS 121187. (**G**,**H**) MFLU 21-0157.] (**I**,**K**) Dry apothecia [(**I**) HKAS 121186. (**K**) HKAS 121188.] (**J**) Margin (HKAS 121186). (**L**,**M**) Concentric ridges [(**L**) MFLU 21-0157. (**M**) HKAS 121182.] (**N**) Compound hairs (MFLU 21-0157). (**P**) Triangular-shaped hairs (MFLU 21-0157). (**O**) Stipe (MFLU 21-0157). (**Q**) Receptacle surface of an apothecium (MFLU 21-0157). Scale bars (**A**,**B**,**E**,**F**) = 5 cm; (**C**,**D**,**G**,**H**) = 3 cm; (**I**–**L**) = 2000 μm; (**M**–**O**) = 1000 μm; (**P**,**Q**) = 500 μm.

**Figure 8:**
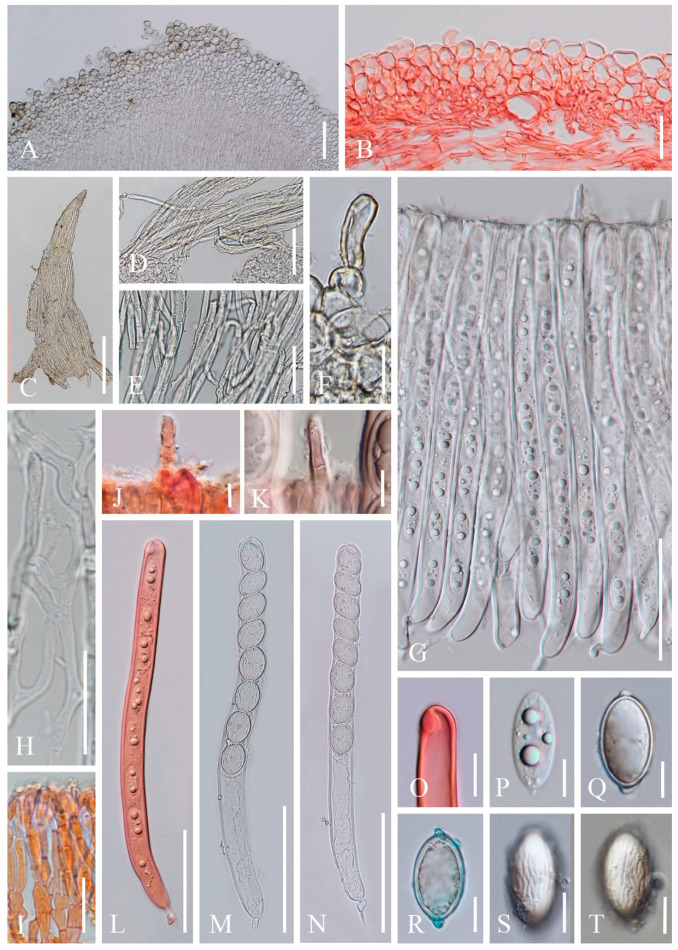
***Cookeina speciosa*** (MFLU 21-0157). (**A**) Vertical section of stipe ectal excipulum. (**B**) Vertical section of receptacle ectal excipulum. (**C**–**E**) Triangular-shaped compound hairs. (**F**) Monilioid process. (**G**) Hymenium including setae from an immature apothecium. (**H**) Paraphyses. (**I**) Apices of paraphyses in CR. (**J**,**K**) Hymenial setae in CR. (**L**) Immature ascus and ascospores in CR from an immature apothecium. (**M**,**N**) Asci and ascospores from a mature apothecium. (**O**) Apex of ascus in CR. (**P**) Immature ascospore. (**Q**–**T**) Mature ascospores. [(**R**) Ascospore in CB.] Scale bars (**A**,**D**,**G**,**N–L**) = 100 μm; (**B**,**F**) = 50 μm. (**C**) = 200 μm; (**E**,**H**,**I**,**O–T**) = 20 μm.

**Figure 9:**
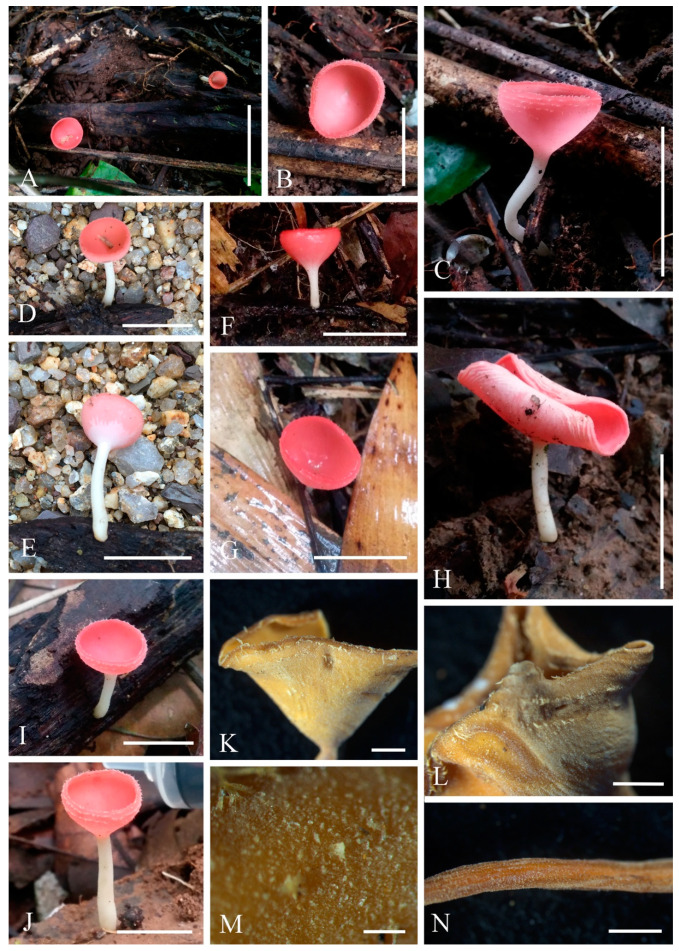
***Cookeina speciosa***. (**A**–**J**) Fresh apothecia [(**A**–**C**) MFLU 21-0158. (**D**,**E**) MFLU 21-0162. (**F**,**G**) MFLU 21-0156. (**H**) MFLU 21-0159. (**I**) MFLU 21-0161. (**J**) MFLU 21-0160. (**K**) Dry apothecium (MFLU 21-0160). (**M**) Receptacle surface of an apothecium (MFLU 21-0160). (**L**) Hairs are arranged in concentric ridges (MFLU 21-0158). (**N**) Stipe (MFLU 21-0158). Scale bars (**A**) = 5 cm; (**B**,**D**–**G**,**I**,**J**) = 2 cm; (**C**,**H**) = 3 cm; (**K**,**L**,**N**) = 2000 μm; (**M**) = 300 μm.

**Figure 10:**
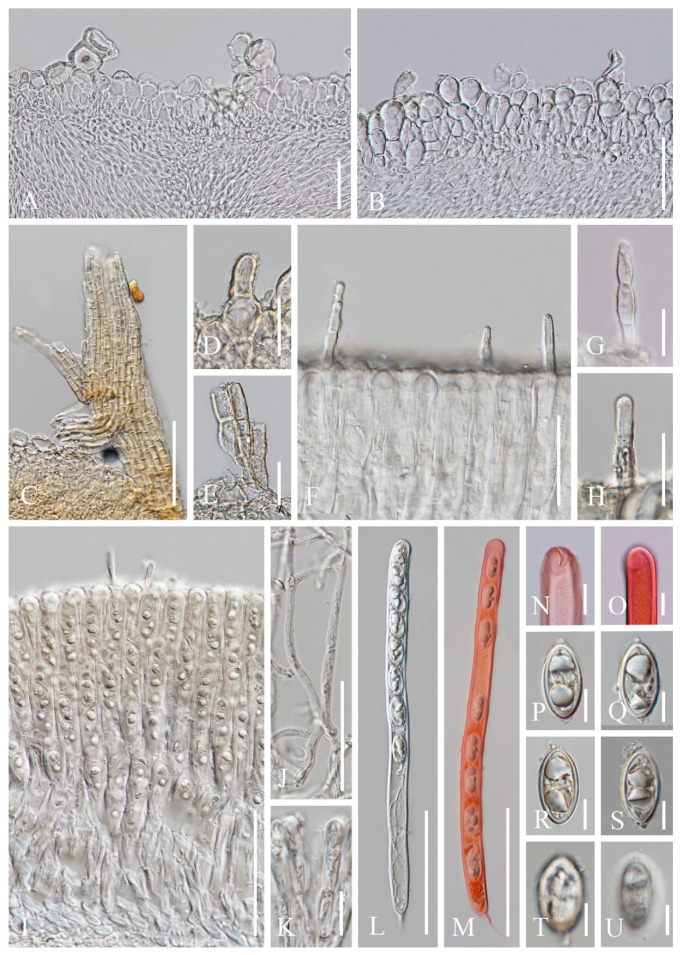
***Cookeina speciosa*** (MFLU 21-0162). (**A**) Vertical section of stipe ectal excipulum. (**B**) Vertical section of receptacle ectal excipulum. (**C**) Triangular-shaped compound hair. (**D**,**E**) Hyphoid hairs. (**F**–**H**) Hymenial setae. (**I**) Hymenium. (**J**) Paraphyses. (**K**) Apices of the paraphyses. (**L**,**M**) Asci and ascospores [(**M**). Ascus and ascospores in CR.] (**N**,**O**) Apical part of asci in CR. (**P**–**U**) Ascospores. Scale bars (**A**,**B**,**F**) = 50 μm; (**C**) = 100 μm; (**D**,**E**,**J**) = 30 μm; (**G**,**H**) = 20 μm; (**K**,**N**–**U**) = 10 μm.

3. ***Cookeina speciosa*** (Fr.) Dennis, Mycotaxon 51: 239 (1994); Figure 7, Figure 8, Figure 9 and Figure 10

Index Fungorum number: IF 362244; Facesoffungi number: FoF 02675

*Saprobic* on dead wood. **Teleomorph**: *Apothecia* up to 4 cm high, up to 3 cm broad, rarely 6 cm broad, solitary or scattered, cupulate, funnel-shaped, usually with a long stipe. *Stipe* up to 2.5 cm long, up to 3 mm broad, central, terete, solid, white to yellowish when fresh, yellow when dry, furfuraceous or tomentose. *Receptacle* cup-shaped, surface yellowish to orange, pale rosy, light ivory (RAL 1015), or pinkish to deep coral, rarely white when fresh, with up to 5 distinct concentric ridges composed of compound hairs, margin entire, or slightly inrolled, with long hairs. *Disc* deeply concave, yellowish to orange or pale rosy to pink when fresh, mostly concolorous with the receptacle surface, or darker in colour. *Stipal ecto-excipulum* 50–140 µm broad, composed of hyaline to yellowish *textura globulosa-angularis*, cells 18–27 × 13–24 μm, some outer globose cells irregularly loosely aggregated forming a pruinose-like surface. *Stipal medulla* composed of hyaline to subhyaline *textura intricata*, hyphae 3–7 μm broad. *Ectal excipulum* 60–160 µm thick, composed of hyaline *textura globulosa-angularis*, cells 17–28 × 12–22 μm, monilioid processes usually composed of 1–2 rounded cells, sometimes with a sub-clavate terminal cell, forming a pruinose-like surface, compound hair bundles up to 800 µm long and up to 260 µm broad at the base, yellow, composed of 5–8 µm wide yellowish septate hyphae, which are fused to triangular-shaped fascicles. *Medullary excipulum* 60–250 μm thick, composed of hyaline *textura intricata*, hyphae interwoven, 2–6 μm wide. *Hymenium* 280–340 μm thick, subhyaline, with hymenial setae, 4–8 μm wide, exceeding the hymenium by 45 μm at most, 1–2 septate, with a rounded apex. *Paraphyses* 2–4 μm wide in the middle part, filiform, septate, mostly constricted at septa, anastomosing to form a network, branched, with a rounded apex. *Asci* 290–320 × 17–21 μm (subclade 4) or 266–300 × 14–17 μm (subclde 5), 8-spored, eccentrically operculate, cylindrical, with obtuse apices and narrow hyphoid base. *Ascospores* [20/1/1, in H_2_O] (24.8–)25.3–27.5(–28.1) × (13.1–)13.7–15(–15.5) μm (Q = 1.69–1.96, **Q** = 1.84 ± 0.07) (subclade 4) or (19.6–)22.2–24.7(–25) × (9.2–)10.3–11.9(–12.3) μm (Q = 1.93–2.38, **Q** = 2.12 ± 0.11) (subclade 5), ellipsoid, rounded at ends, uniseriate, equilateral, biguttulate when mature, immature multiguttulate, projecting apiculi present at one or both poles, perispore ornamented with anastomosing cyanophobic longitudinal striae. **Anamorph**: not seen.

Material examined: CHINA, YUNNAN, XISHUANGBANNA: on unidentified dead branch under broadleaved forest, 5 June 2018, Ming Zeng, Zeng003 (HKAS 121180), Zeng004 (HKAS 121181); ibid., 6 June 2018, Ming Zeng, Zeng006 (HKAS 121182), Zeng007 (HKAS 121183), Zeng008 (HKAS 121184); ibid., 12 June 2018, Ming Zeng, Zeng023 (HKAS 121185); ibid., 27 August 2019, Ming Zeng, ZM356 (HKAS 124640); Mengla, on unidentified dead branch under broadleaved forest, 27 August 2019, Ming Zeng, ZM332 (HKAS 121186), ZM336 (HKAS 121187); ibid., on unidentified trunk under broadleaved forest, ZM339 (HKAS 121188); THAILAND: Phangnga, Kapong, on unidentified dead branch under broadleaved forest, 29 August 2017, Chuangen Lin, 4-1 (MFLU 21-0157); ibid., Thap Phut, on unidentified dead branch under broadleaved forest, 30 August 2017, Chuangen Lin, 5-1 (MFLU 21-0158), 5-3 (MFLU 21-0159), 5-5 (MFLU 21-0160); ibid., 1 September 2017, Chuangen Lin, 11-1 (MFLU 21-0161); Prachuap Khiri Khan, Bang Saphan, on unidentified dead branch under broadleaved forest, 28 August 2017, Chuangen Lin, 1-1 (MFLU 21-0156); Ranong, on unidentified dead branch under broadleaved forest, 7 October 2017, Ming Zeng, ST09 (MFLU 21-0162).

Notes: This species is widely distributed in tropical areas [67]. The main feature of this species is the variable colour of the apothecia with up to five distinct concentric ridges close to margin, and compound hairs arranged on these ridges. In addition, hymenium has hymenial setae, broadly ellipsoid ascospores with obviously projecting apiculi, and longitudinal striae on surface of the ascospores, anastomosed in some parts [48,67]. This species was introduced by Dennis [72] based on *Peziza speciosa* Fr.; meanwhile, *C. sulcipes* was synonymized as a later epithet [72]. Although phylogenetic studies based on ITS show some genetic variation associated with colour differences within the *C. speciosa* clade [48], Iturriaga and Pfister [67] still consider this as a complex. In this study, *Cookeina garethjonesii* represented by two strains formed an independent branch, which is nested in the *C. speciosa* clade in subclade 4 (Figure 1). The phylogeny herein contains a comprehensive sampling of *C. speciosa* sequences. *Cookeina garethjonesii* was established as a separate species from *C. speciosa* due to the lack of hymenial setae and smooth-walled ascospores [50]. In the illustration of the holotype of *C. garethjonesii* provided in the original study, distinct hymenial setae are clearly visible [50] (Figure 2 in the original paper). In their phylogeny, the species was phylogenetically distinct; however, only a limited number of *C. speciosa* sequences were used in the dataset. Based on these contradictions, we suggest that the type specimen should be re-examined in the future. In our trees, six collections were grouped with a sequence designated as *C. sulcipes* (MFLU 15-2358), forming a distinct clade within *C. speciosa* complex (subclade 5, Figure 1). This well-defined clade has members that produce almost uniformly pink to coral apothecia (Figure 9). However, the coral-coloured apothecia are not unique to subclade 5; rather, collections with coral apothecia are spread across *C. speciosa* complex (e.g., FH Iturriaga 1E-D5, FH Iturriaga 4A-D4 and FH Iturriaga 7A-D4 from the subclades 1 and 3, Figure 1) [48] (this study). In our described collections, *C. speciosa* (MFLU 21-0157) (Figure 8) from subclade 4 has broader asci (17–21 μm vs. 14–17 μm) and larger ascospores (25.3–27.5 × 13.7–15.0 μm vs. 22.2–24.7 × 10.3–11.9 μm) when compared to *C. speciosa* (MFLU 21-0162) (Figure 10) from subclade 5. Nevertheless, *C. sulcipes* (MFLU 15-2358) described by Ekanayaka et al. [50] has an indistinguishable size of asci (280–380 × 15–22 μm) and ascospore (21–30 × 11–18 μm) compared to both our described collections. Hence, it seems that the sizes of asci and ascospores cannot distinguish these specimens at the species level. Within the *C. speciosa* complex, our collections placed in two subclades, both of which have high statistical support (subclade 4: 87BS/0.99PP, subclade 5: 97BS/1.00PP). More sampling and type studies are needed to resolve *C. speciosa* complex in the future.

**Figure 11:**
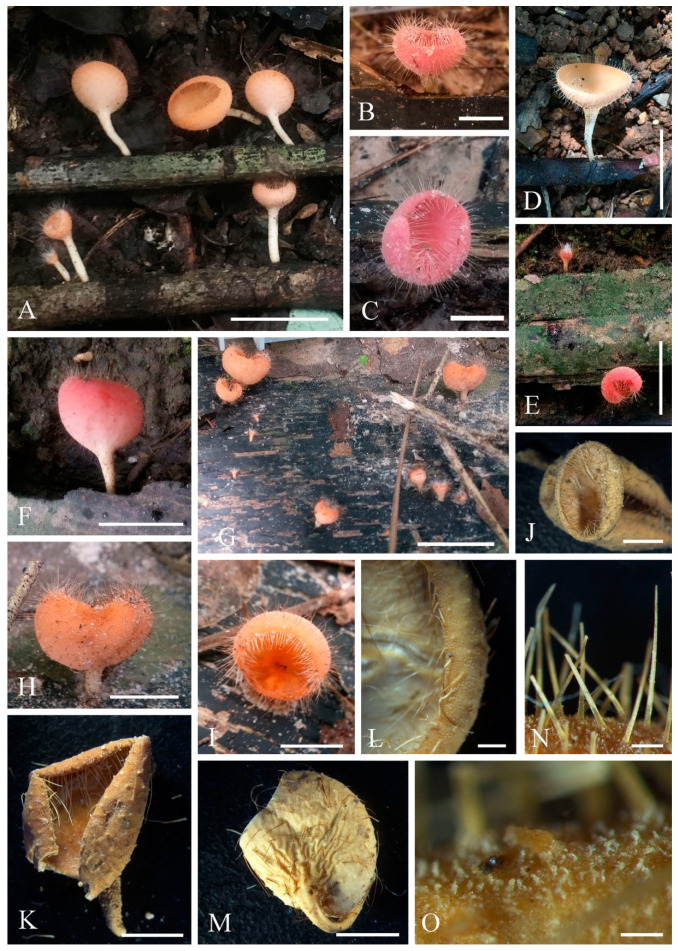
***Cookeina tricholoma***. (**A**–**I**) Fresh apothecia [(**A**) HKAS 121191. (**B**,**C**) MFLU 21-0165. (**D**) MFLU 21-0163. (**E**,**F**) MFLU 21-0168. (**G**–**H**) MFLU 21-0167. (**I**) MFLU 21-0166.] (**J**,**K**,**M**) Dry apothecia [(**J**) MFLU 21-0168. (**K**) MFLU 21-0165. M HKAS 121191.] (**L**) Margin (HKAS 121191). (**N**) Compound hairs (MFLU 21-0164). (**O**) Receptacle surface of an apothecium (MFLU 21-0163). Scale bars (**A**,**E**,**G**) = 3 cm; (**B**,**C**,**H**,**I**) = 1 cm; (**D**,**F**) = 2 cm; (**J**,**K**,**M**) = 5000 μm; (**L**) = 1000 μm; (**N**) = 500 μm; (**O**) = 200 μm.

**Figure 12:**
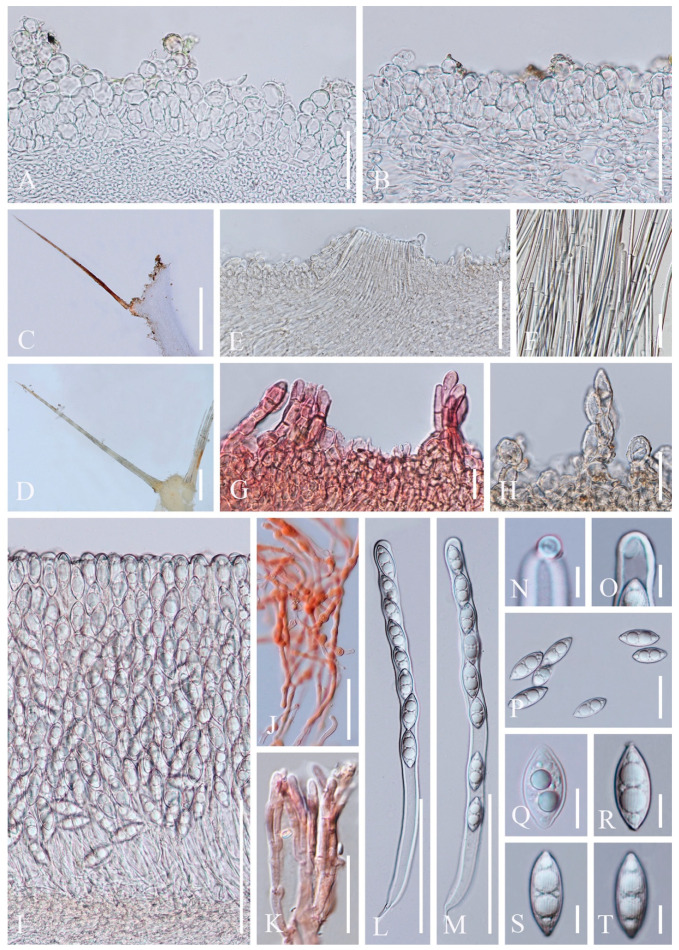
***Cookeina tricholoma***. (**A**) Vertical section of stipe ectal excipulum. (**B**) Vertical section of receptacle ectal excipulum. (**C**,**D**) Compound hairs. (**E**) Broken compound hair from medullary excipulum. (**F**) Loose compound hair. (**G**) Hyphoid hairs in CR. (**H**) Monilioid processes. (**I**) Hymenium. (**J**) Paraphyses in CR. (**K**) Apices of paraphyses in CR. (**L**,**M**) Asci and ascospores. (**N**,**O**) Apices of the asci. (**P**) Ascospores. (**Q**) Ascospore when young. (**R**–**T**) Ascospores ornamented by fine longitudinal striate ridges when mature. Scale bars (**A**,**B**,**F**) = 50 μm; (**C**,**D**) = 500 μm; (**E**,**I**,**L**,**M**) = 100 μm; (**G**,**H**,**J**,**K**) = 20 μm; (**N**–**T**) = 10 μm.

4. ***Cookeina tricholoma*** (Mont.) Kuntze, Revis. gen. pl. (Leipzig) 2: 849 (1891); Figure 11 and Figure 12

Index Fungorum number: IF 121551; Facesoffungi number: FoF 02677

*Saprobic* on dead wood. **Teleomorph**: *Apothecia* up to 3 cm high, up to 2 cm broad, solitary or scattered, cupulate, fleshy, with short to long stipe. *Stipe* up to 1.5 cm long, up to 2 mm broad, central, rarely eccentrical, terete, solid, yellowish or pinkish when fresh, yellow when dry, furfuraceous or tomentose, with long compound hairs as the receptacle. *Receptacle* cup-shaped, surface yellowish to orange, or pink when fresh, with long compound hairs, tomentose, margin entire, rarely deeply split on one side, inrolled when dry. *Disc* deeply concave, yellow to orange, or pinkish when fresh, mostly concolorous with the receptacle surface. *Stipal ecto-excipulum* 85–175 µm broad, composed of hyaline to yellowish *textura globulosa-angularis*, cells 12–17 × 11–15 μm, some outer globose cells irregularly loosely aggregated forming a pruinose-like surface. *Stipal medulla* composed of hyaline *textura intricata*, hyphae 5–7.5 μm broad. *Ectal excipulum* 50–80 µm thick, composed of hyaline *textura globulosa-angularis*, cells 13–19 × 11–15 μm, with two types of hairs mixed throughout the ectal excipular surface: fasciculate hyphoid hairs, composed of 5–9 µm wide subhyaline to yellowish, septate, broad hyphae; additionally with monilioid processes composed of 2 or more globose cells forming a pruinose-like surface. *Medullary excipulum* 100–160 μm broad, composed of hyaline *textura intricata*, hyphae interwoven, 3–5 μm wide. *Compound hairs* up to 5 mm long, up to 200 µm diameter at base of fascicle, fasciculate, brown, arising from the medullary excipulum, composed of 3–6 µm diameter, parallel, yellowish, septate, thick-walled hyphae, with a rounded end. *Hymenium* 280–350 μm thick, subhyaline. *Paraphyses* 1.5–3 μm broad in the middle part, filiform, septate, anastomosing to form a network, branched, with a rounded end. *Asci* 308–342 × 13–21 μm, 8-spored, eccentrically operculate, cylindrical, with obtuse apices and narrow bases. *Ascospores* [20/1/1, in H_2_O] (25.8–)28.0–32.9(–33.9) × (11–)11.6–12.8(–13.8) μm (Q = 2.33–2.68, **Q** = 2.50 ± 0.17), fusiform, pointed at ends, uniseriate, equilateral, biguttulate to multiguttulate, projecting apiculi sometime present at one pole, ornamentation with fine longitudinal striate ridges. **Anamorph**: not seen.

Material examined: CHINA, YUNNAN, XISHUANGBANNA: on unidentified dead branch under broadleaved forest, 5 June 2018, Ming Zeng, Zeng002 (HKAS 121189), Zeng005 (HKAS 121190); ibid., 26 August 2019, Ming Zeng, ZM328 (HKAS 121191). THAILAND: Chiang Rai, Song Khwae, on unidentified dead branch under broadleaved forest, 13 August 2017, Ming Zeng, N003 (MFLU 21-0163); Ranong, on unidentified dead branch under broadleaved forest, 7 October 2017, Ming Zeng, ST10 (MFLU 21-0164); Phangnga, Thap Phut, on unidentified dead branch under broadleaved forest, 30 August 2017, Chuangen Lin, 6-1 (MFLU 21-0165); ibid., 1 September 2017, Chuangen Lin, 11-2 (MFLU 21-0168), 11-3 (MFLU 21-0169); ibid., on unidentified dead trunk under broadleaved forest, Chuangen Lin, 6-2 (MFLU 21-0166), 6-3 (MFLU 21-0167).

Notes: This species is distinguished by yellow to orange, or coral apothecia with long compound hairs extending from medullary excipulum, fusiform ascospores with longitudinal striae [67]. It is similar to *C. korfii* and *C. sinensis*, both of which have smooth-walled ascospores [52]. This species is widely distributed in tropical areas and is also a common species in southwest China and Thailand [50,67].

**Figure 13:**
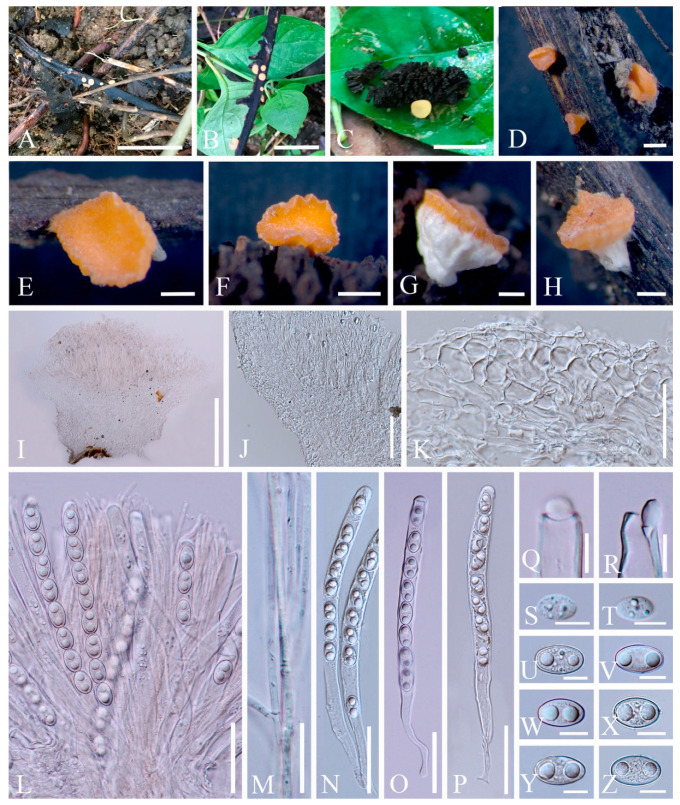
***Nanoscypha aequispora*** (MFLU 21-0170, holotype). (**A**–**H**) Apothecia. (**I**) Vertical median section of apothecia. (**J**) Vertical median section of flank. (**K**) Vertical section of receptacle ectal excipulum. (**L**) Hymenium. (**M**) Paraphyses. (**N**–**P**) Asci and ascospores. (**Q**–**R**) Asci apices. (**S**,**T**) Immature ascospores. (**U**–**Z**) Ascospores when mature. Scale bars (**A**) = 2 cm; (**B**) = 1 cm; (**C**) = 5 mm; (**D**,**F**) = 1000 μm; (**E**,**G**–**I**) = 500 μm; (**J**) = 100 μm; (**K**,**L**,**N**–**P**) = 50 μm; (**M**) =20 μm; (**Q**–**Z**) =10 μm.

5. ***Nanoscyphaaequispora*** M. Zeng, Q. Zhao & K.D. Hyde, sp. nov.; Figure 13

Index Fungorum number: IF 559928; Facesoffungi number: FoF 10410

Etymology: The specific epithet refers to equilateral ascospores.

Holotype: MFLU 21-0170

Diagnosis: This species is diagnosed by turbinate to shallowly cupulate apothecia with broadly whitish stipe, yellowish to orange disc, undulate margin, filiform paraphyses with yellowish granules, ellipsoid and equilateral ascospores with biguttulate.

*Saprobic* on dead wood and plant fruit. **Teleomorph**: *Apothecia* 0.5–1 mm high, 1–2 mm broad, scattered, shallowly cupulate when fresh, turbinate when dry, broadly stipitate, glabrous. *Stipe* 400–1500 µm long, 500–2000 µm broad, central, funnel-shaped, wrinkled on surface, solid, whitish to cream, rarely yellowish. *Receptacle* shallowly cupulate, surface yellowish to orange, margin undulate. *Disc* shallowly concave to discoid, concolorous with the receptacle surface. *Stipal ecto-excipulum* 62–166 µm, composed of hyaline *textura angularis*, cells 14–20 × 8–12 μm, mixed with *textura prismatica*, cells 18–25 × 9–12 µm, with some porrectoid cells arranged on surface, cells 4–6 µm wide. *Stipal medulla* composed of hyaline *textura intricata*, hyphae 4–6 μm broad. *Ectal excipulum* 56–94 µm thick, composed of hyaline to yellowish *textura globulosa-angularis*, cells 14–22 × 9–13 μm, mixed with *textura prismatica*, cells 19–25 × 9–13 µm, with some porrectoid cells arranged on surface, cells 4–6 µm wide. *Medullary excipulum* 76–192 µm thick, composed of hyaline *textura intricata*, hyphae 4–6 µm wide. *Hymenium* 280–310 µm thick, yellowish, paraphyses slightly exceeding the asci when dehydrated. *Paraphyses* 2–3 µm wide in the middle part, filiform, branched, septate, filled with yellowish granules. *Asci* 235–284 × 10–13 µm, 8-spored, subterminally operculate, apices obtuse, cylindrical, becoming narrow towards the base. *Ascospores* [20/1/1, in H_2_O] (14.8–)16.2–18.6(–19.2) × (12.4–)10.3–11.6(–9.9) µm (Q = 1.41–1.78, **Q** = 1.59 ± 0.14), ellipsoid, with round or slightly truncated ends, equilateral, rarely slightly inequilateral with one side flat, uniseriate, multiguttulate when immature, biguttulate when mature, smooth-walled. **Anamorph**: not seen.

Material examined: THAILAND: Ranong, on unidentified twigs and plant fruit, 7 October 2017, Ming Zeng, ST07 (MFLU 21-0170, holotype); ibid., 8 October 2017, Ming Zeng, ST11 (MFLU 21-0171, paratype).

Notes: Species in the genus *Nanoscypha* are small cup-fungi, normally less than 1 cm in diameter. Apothecia vary from discoid to cupulate, to turbinate or funnel-shaped, sessile to stipitate, with yellow, orange to red in colour. Cylindrical asci 3-, 4-, 6-, or 8-spored, and having tapered bases. Ascospores are ellipsoid to reniform, mostly inequilateral, rarely equilateral, mostly with two oil drops [6,73]. There are currently eight species assigned in this genus according to Species Fungorum [27], while the placement of *N. striatispora* (W.Y. Zhuang) F.A. Harr. [6,74] is still under debate (see discussion). Although our new species and *N. tetraspora* clustered together, it is difficult to confirm the correct position of new species in the phylogenetic tree due to the lack of available data for other species. Meanwhile, *Nanoscypha* strains are nested inside a clade together with *Phillipsia* and *Rickiella* in the ITS and multigene analyses. Through morphological comparison (Table 2), most species share inequilateral ascospores, except for *N. macrospora* Denison [6,74,75] and two vaguely-described species, *N. bella* (Berk. & M.A. Curtis) Pfister and *N. euspora* (Rick) S.E. Carp. Of these three, *N. bella* has larger-sized apothecia and ascospores [76], while *N. euspora* differs in its uniguttulate ascospores [77]. *Nanoscypha macrospora* is having equilateral ascospores, rarely inequilateral, same as our new species. The main difference of the *N. macrospora* is that the asci contain only 3 or 4 ascospores. In addition, the ascospores are elongated ellipsoid in shape [73]. Moreover, *N. orissaensis* C.M. Das & D.C. Pant is a rarely-recorded species, which lacks type material [75]. Thus, we proposed the new species *Nanoscypha aequispora* here based on morphology.

**Figure 14:**
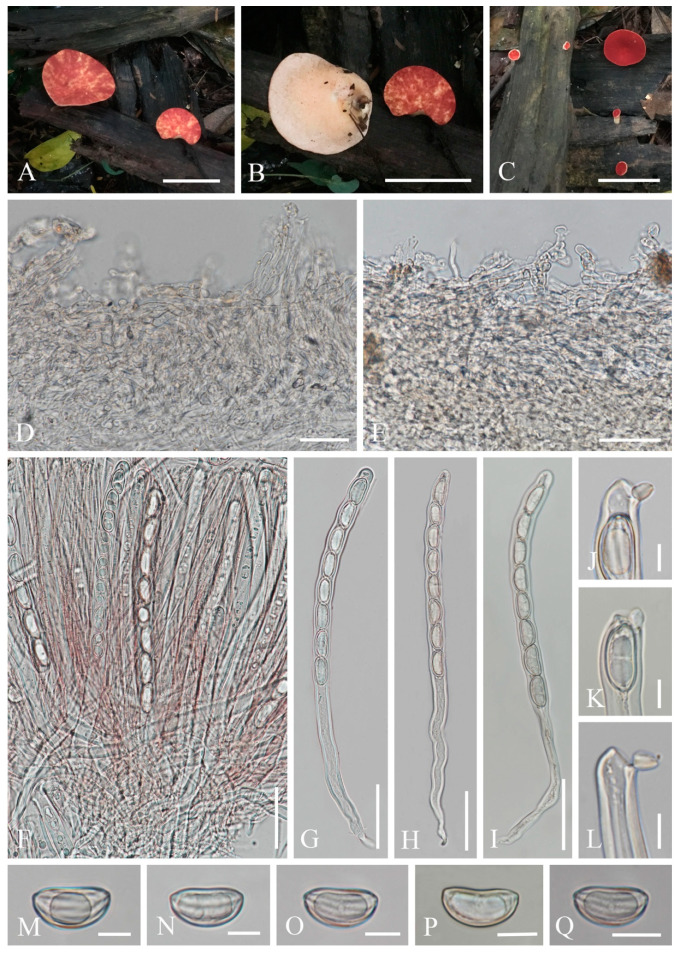
***Phillipsia domingensis***. (**A**–**C**) Fresh apothecia [(**A**,**B**) HKAS 121193. (**C**) HKAS 121192.] (**D**) Vertical section of stipal ecto-excipulum. (**E**) Vertical section of ectal excipulum. (**F**) Hymenium. (**G**–**I**) Asci and ascospores. (**J**–**L**) Asci apices. (**M**–**Q**) Ascospores. Scale bars (**A**–**C**) = 3 cm; (**D**,**E**) = 30 μm; (**F**–**I**) = 50 μm; (**J**–**Q**) = 10 μm.

6. ***Phillipsia domingensis*** (Berk.) Berk. ex Denison, Mycologia 61(2): 293 (1969); Figure 14

*=Phillipsia gelatinosa* Ekanayaka, Q. Zhao & K.D. Hyde, Phytotaxa 316(2): 142 (2017)

Index Fungorum number: IF 122362; Facesoffungi number: FoF 02868

*Saprobic* on dead wood. **Teleomorph**: *Apothecia* 7–11 mm high, up to 4 cm broad, scattered, leathery, shallowly cupulate to discoid, substipitate to shortly stipitate. *Stipe* up to 3 mm long, 5 mm broad, central to eccentrical, obconical, solid, bright beige red (RAL 3012), or reddish, or creamy yellowish, pubescent. *Receptacle* shallowly cupulate, surface concolorous with the stipe, pubescent, margin entire. *Disc* shallowly cup-shaped to discoid, pearl pink (RAL 3033) to orient red (RAL 3031), or with yellow patches. *Stipal ecto-excipulum* 60–90 µm thick, composed of yellowish to subhyaline *textura porrecta* to *textura epidermoidea*, hyphae 4–7 µm wide, with some outermost hyphae irregularly loosely aggregated to form pubescent surface. *Stipal medulla* composed of hyaline *textura intricata*, hyphae 3.5–5 μm broad. *Ectal excipulum* 60–100 µm thick, composed of yellowish to subhyaline *textura porrecta* to *textura epidermoidea*, hyphae 4–7 µm wide, with some loose hyphae in the outermost part. *Medullary excipulum* 280–650 µm thick, composed of hyaline *textura intricata*, hyphae 3–4 µm wide. *Hymenium* 300–350 µm thick, pink to red, paraphyses slightly exceeding the asci when dehydrated. *Paraphyses* 1–2 µm broad in the middle part, filiform, with reddish contents, septate, branched. *Asci* 285–346 × 11–14 µm, 8-spored, eccentrically operculate, cylindrical, apices obtuse, becoming narrow towards the base. *Ascospores* [20/1/1, in H_2_O] (19.8–)20.5–24.2(–27.7) × (10.4–)10.6–12(–13.2) µm (Q = 1.82–2.17, **Q** = 1.98 ± 0.08), subreniform or reniform with pointed or subpapillate ends, uniseriate, inequilateral, uniguttulate or biguttulate, ornamented with several longitudinal striations. **Anamorph**: not seen.

Material examined: CHINA, YUNNAN, XISHUANGBANNA: on unidentified dead branch under broadleaved forest, 26 August 2019, Ming Zeng, ZM329 (HKAS 121192), ZM330 (HKAS 121193). THAILAND, CHIANGMAI: on rotten wood, 18 June 2016, H. Maoqiang, LE2016112 (MFLU 16-2992); Mushroom Research Center (MRC), on rotten wood, 12 December 2015, S.C. Karunarathna, HD044 (MFLU15-2360, holotype? of *Ph. gelatinosa*); ibid., 12 June 2016, A.H. Ekanayaka, HD057 (MFLU 16-2956, holotype? of *Ph. gelatinosa*).

**Figure 15:**
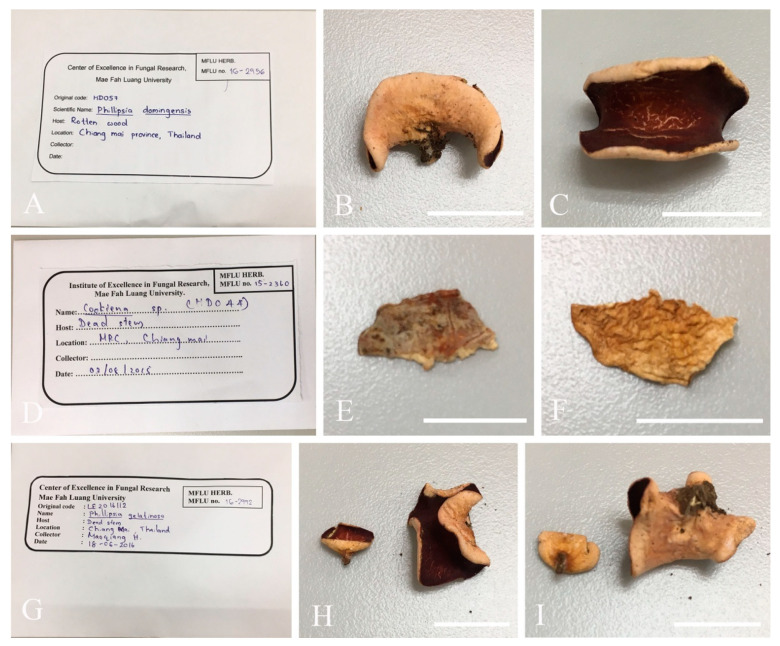
Herbarium materials of *Phillipsia gelatinosa*. (**A**–**C**) MFLU 16-2956 (? holotype). (**D**–**F**) MFLU 15-2360 (? holotype). (**G**–**I**) MFLU 16-2992. Scale bars (**B**,**C**,**E**,**F**,**H**,**I**) = 2 cm.

**Figure 16:**
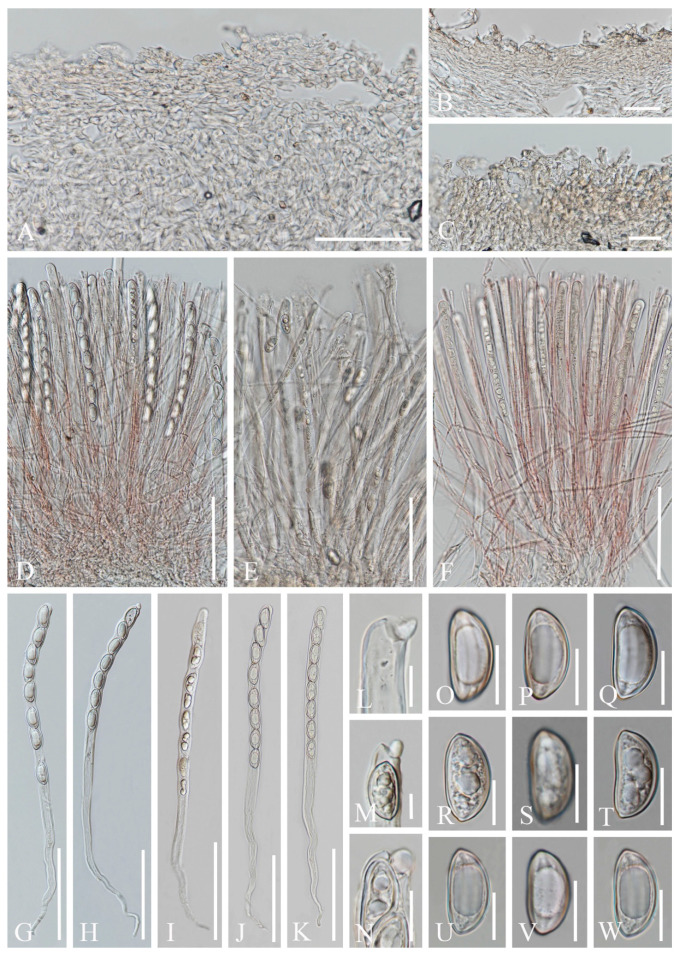
Sections of herbarium materials of *Phillipsia gelatinosa*. (**A**,**F**,**J**,**K**,**N**,**U**–**W**) MFLU 16-2992. (**B**,**E**,**I**,**M**,**R**–**T**) MFLU 15-2360. (**C**,**D**,**G**,**H**,**L**,**O**–**Q**) MFLU 16-2956. (**A**–**C**) Vertical section of receptacle ectal excipulum. (**D**–**F**) Hymenium. (**G**–**K**) Asci and ascospores. (**L**–**N**) Apices of asci. (**O**–**W**) Ascospores. Scale bars (**A**) = 50 μm; (**B**,**C**) = 30 μm; (**D**–**K**) = 100 μm; (**L**–**N**) = 10 μm; (**O**–**W**) = 15 μm.

**Figure 17:**
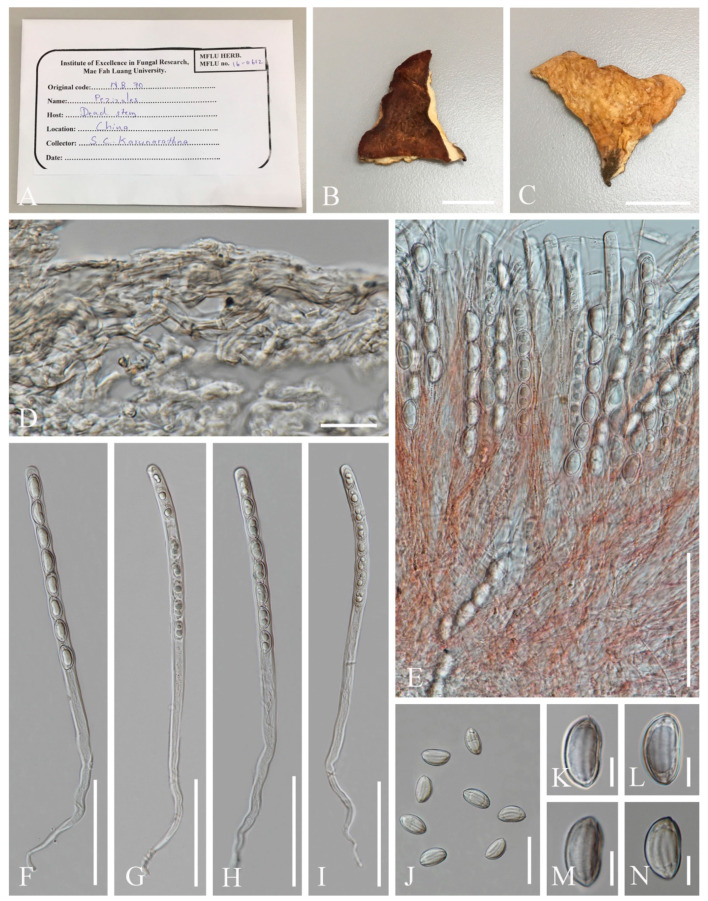
***Phillipsia subpurpurea*** (MFLU16-0612). (**A**–**C**) Herbarium materials. (**D**) Vertical section of receptacle ectal excipulum. (**E**) Hymenium. (**F**–**I**) Asci and ascospores. (**J**–**N**) Ascospores. Scale bars (**B**,**C**) = 1 cm; (**D**) = 20 μm; (**E**–**I**) = 100 μm; (**J**) = 40 μm; (**K**–**N**) = 10 μm.

Notes: This is a common species in the subtropical and tropical areas. This species has larger-sized apothecia, red to purple-red hymenium, subreniform or reniform ascospores with several conspicuous longitudinal striations [57,80]. Hansen et al. [53] suggested the *Ph. domingensis* complex based on ITS genetic marker, owing to the species *Ph. lutea* Denison and some *Ph. domingensis* collections featuring yellow apothecia, which nested in the typically red *Ph. domingensis*. Ekanayaka et al. [58] introduced *Ph. gelatinosa* based on three collections and provided a description of *Ph. subpurpurea* Berk. & Broome along with molecular data, which didn’t exist before. Even these two species are placed in the *Ph. domingensis* complex, Ekanayaka et al. [58] identified morphological differences to distinguish them from *Ph. domingensis*. *Phillipsia gelatinosa* is distinguished by its orange contents of paraphyses, larger-sized asci and ascospores (Table 3), and presence of a gelatinous sheath surrounding ascospores [58]. *Phillipsia subpurpurea* (MFLU16-0612) differs in that has smooth ascospores or with faint striations, a thick gelatinous sheath surrounding ascospores. We re-examined all specimens named *Ph. gelatinosa* and *Ph. subpurpurea* (MFLU16-0612) (Figure 15, Figure 16 and Figure 17). Three of them show morphological features almost consistent with *Ph. domingensis*, which have distinct reddish contents in paraphyses, subreniform or reniform ascospores with conspicuous longitudinal striations. Although conspicuous reddish contents and striate ascospores are difficult to observe in *Ph. gelatinosa* (MFLU 15-2360) due to the quality of the specimen, we still can find sporadic reddish contents in paraphyses and faint striations on surface of ascospores. For all specimens, sheath-like structure was only present when rehydrating in 10% KOH, not in water. By comparing the sizes of asci and ascospores of the re-examined specimens (Table 3), there are no significant differences among these specimens, while these sizes are largely different from Ekanayaka et al.’s descriptions. Most importantly, the “two holotypes” (MFLU 16-2956 and MFLU 15-2360) were assigned in the protologue of *Ph. gelatinosa* [58]. According to the International Code of Nomenclature for algae, fungi, and plants, specifically the Art. 8.1. in Shenzhen Code [81], *Ph. gelatinosa* is an invalid name. Based on these, *Ph. gelatinosa* should be a *nomen invalidum* and a synonym of *Ph. domingensis*. Meanwhile, the specimen (MFLU16-0612) was incorrectly assigned to *Ph. subpurpurea* which should be corrected to *Ph. domingensis*. *Phillipsia domingensis* is a complex lacking type sequences with almost solely ITS region known for most collections. Thus, for efficient differentiation at the species level, sequencing of additional DNA regions and more data on phenotypic characters of as many collections possible are needed.

**Figure 18:**
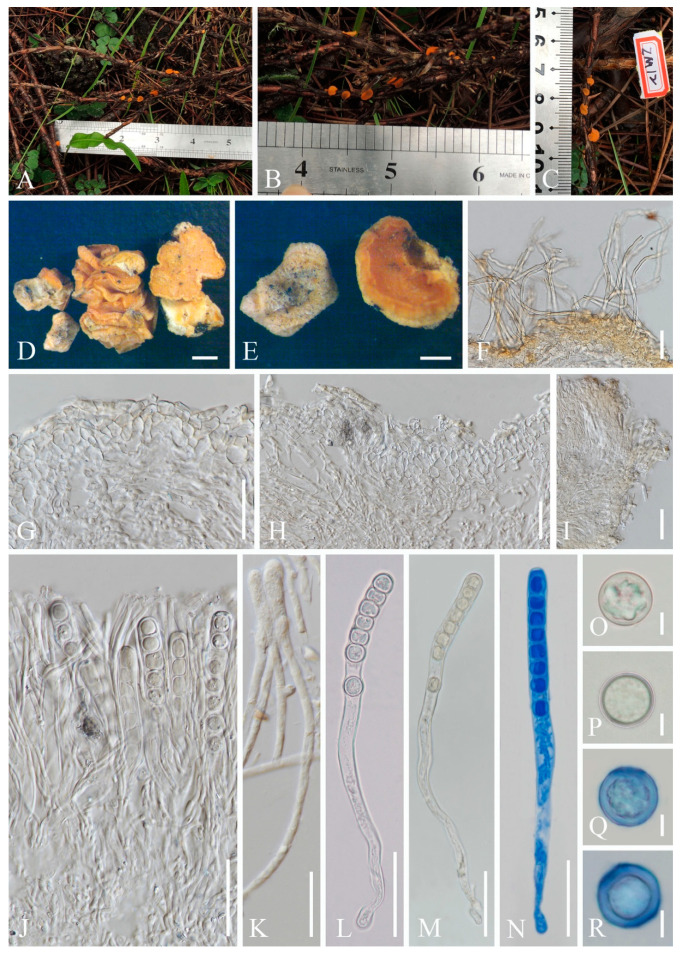
***Pithyavillosa*** (HKAS 104653, holotype). (**A**–**C**) Fresh specimens. (**D**,**E**) Dry specimens. (**F**) Hyphoid hairs on the surface of receptacle ectal excipulum. (**G**,**H**,**I**) Vertical section of receptacle ectal excipulum on the upper flank. (**J**) Hymenium. (**K**) Paraphyses. (**L**–**N**) Asci and ascospores [(**N**) Ascus and ascospores in CB.] (**O**–**R**) Ascospores (**Q**,**R**) Ascospore in CB. Scale bars (**D**) = 1000 μm; (**E**) = 500 μm; (**F**–**J**,**L**–**N**) = 50 μm; (**K**) = 20 μm; (**O**–**R**) = 5 μm.

7. ***Pithya villosa*** M. Zeng, Q. Zhao & K.D. Hyde, sp. nov.; Figure 18

Index Fungorum number: IF 559929; Facesoffungi number: FoF 10411

Etymology: The specific epithet refers to villose receptacle surface.

Holotype: HKAS 104653

Diagnosis: This species is diagnosed by shallowly cupulate to discoid, or convex apothecia growing on *Juniperus* sp., yellowish excipular surface covered with hyphoid hairs, entire or lobate margin, subhyaline to yellowish paraphyses, spherical ascospores with granular contents.

*Saprobic* on twigs of *Juniperus* sp. **Teleomorph**: *Apothecia* 2–3 mm high, 3–6 mm broad, scattered to gregarious, fleshy, shallowly cupulate to discoid, or convex, sessile to substipitate. *Receptacle* shallowly cupulate, margin entire to lobate when fresh, or curled when dry, subglabrous to finely pubescent, whitish, flanks pubescent to villose towards the base, whitish on yellowish ground. *Disc* discoid to slightly convex, yellow to orange. *Ectal excipulum* 60–100 µm broad, hyaline on a wider marginal area, composed of *textura porrecta*, subhyaline to yellowish towards the base, and composed of *textura epidermoidea* with cells 5–8 µm broad, to *textura angularis*, with cells 13–17 × 9–12 µm. Hairs mostly arise from the excipular flank surface and apothecial base, subhyaline, flexuous, hyphoid, septate, 5–7 µm wide. *Medullary excipulum* 60–210 µm broad, of *textura intricata*, hyaline, composed of 4–6 µm broad hyphae. *Hymenium* 180–290 µm thick, yellow, paraphyses slightly exceeding the asci when dehydrated. *Paraphyses* 2–3 µm broad in the middle part, filiform, apex enlarged, 4–6 µm broad, branched, septate, subhyaline to yellowish. *Asci* 227–275 × 11–14 µm, 8-spored, terminally operculate, subcylindrical, apex obtuse, becoming narrow towards the base. *Ascospores* [20/1/1, in H_2_O] (11.4–)11.7–13.7(–14.4) × (11.3–)11.7–13.8(–14.0) µm (Q = 0.93–1.14, **Q** = 1.00 ± 0.05), spherical, uniseriate, subhyaline, with refractive granular contents, smooth-walled. **Anamorph**: not seen.

Material examined: CHINA: Yunnan, Shangri-La, on twigs of *Juniperus* sp., elev. 3413 m a.s.l., 14 August 2018, Ming Zeng, ZM12 (HKAS 104653, holotype); ibid. elev. 3390 m a.s.l., 15 August 2018, Ming Zeng, ZM23 (HKAS 121194, paratype).

Notes: *Pithya* is unique in *Sarcoscyphaceae* with its spherical ascospores [73]. Most species have similar features, namely yellow to orange apothecia, shallowly cupulate to discoid, sessile to substipitate, filiform paraphyses with enlarged apices, and smooth-walled spherical ascospores [73,82,83]. Kirk et al. [84] and Wijayawardene et al. [16,85] respectively accounted five and two species in this genus without listing species names, while there are 11 species records in Species Fungorum [27], excluding *Pi. thujina* (Peck) Sacc. which is synonymised to *Pi. cupressina* [82]. To our knowledge, the species richness does not appear to be clarified in this genus. Our introduction of the new species is based on a comparison of ten *Pithya* species according to Species Fungorum records. *Pithya cupressina* and *Pi. vulgaris* Fuckel are the oldest two species introduced in this genus, and they are also the most commonly recorded [73,82,83,86,87,88,89,90,91,92,93]. Molecular data are also most abundant in these two species [15,21,59]. *Pithya vulgaris* has larger apothecia and ascospores than *Pi. cupressina*, and whereas *Pi. vulgaris* mainly grows on the dead branches of *Abies* or *Pinus*, *Pi. cupressina* commonly grows on *Cupressus* or *Juniperus* [82,90,91]. Although our species is also found from *Juniperus*, it differs from *Pi. cupressina* in its apothecial vesture. While our new species has villose surface where hyphoid hairs are covering the whole excipular surface with margin, and to some extent also wider marginal area, *Pi. cupressina* has smooth excipular surface and white hyphal hairs are only present at the base [6,73,82,90]. The new species is phylogenetically distinct and is sister to *Pi. cupressina*. For other species that are rarely described, most of them are collected from Pinaceae with limited number of records, such as *Pi. arctica* L.I. Vassiljeva [94], *Pi. epichrysea* (Beck) Boud. [95], *Pi. lacunosa* (Ellis & Everh.) Seaver [82], *Pi. malochi* Velen. and *Pi. microspora* Velen. [96]. *Pi. arethusa* Velen. was collected from *Ligustrum vulgare* [96] and *Pi. madothecae* Buchloh was collected from *Porella platyphylla* [97]. *Pithya fascicularis* (Berk. & Broome) Sacc. was only described from bark, but its subglobose and small-sized ascosposes (7–8 µm diameter) is enough to distinguish it from our new species [95].

**Figure 19:**
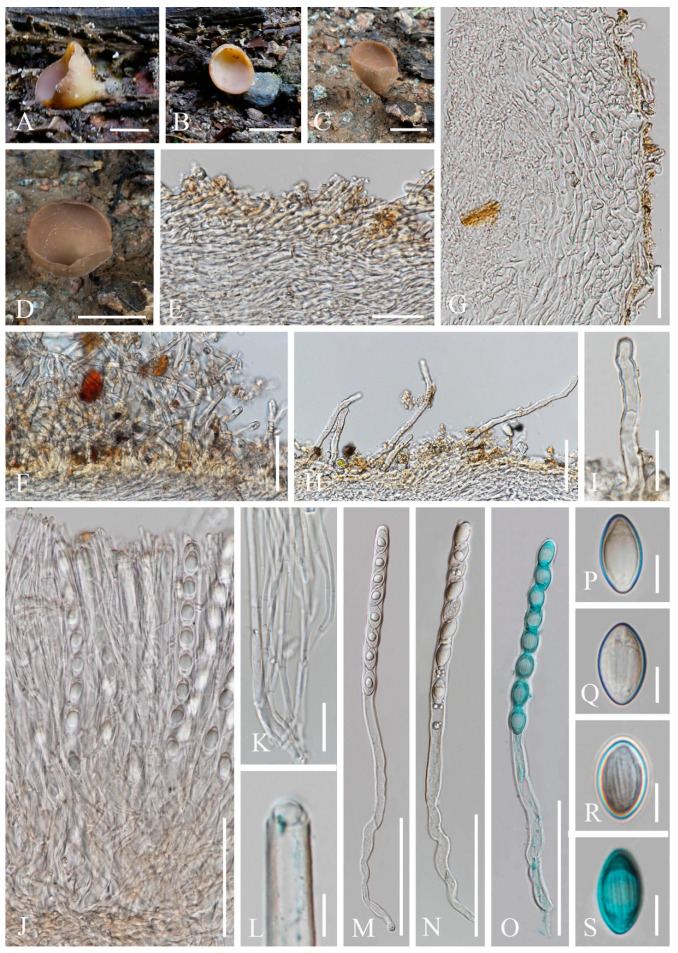
***Sarcoscyphalongitudinalis***. (**A**–**D**) Fresh specimens [(**A**,**B**) HKAS 121196. (**C**,**D**) HKAS 121195.] (**E**) Vertical section of stipe ectal excipulum. (**F**) Hyphoid hairs on the base. (**G**) Vertical section of receptacle ectal excipulum. (**H**,**I**) Hyphoid hairs from receptacle ectal excipulum. (**J**) Hymenium. (**K**) Paraphyses. (**L**) Apex of ascus. (**M**–**O**) Asci and ascospores [(**O**) Ascus and ascospores in CB.] (**P**–**S**) Ascospores [(**S**) Ascospore in CB.] Scale bars (**A**) = 1 cm; (**B**–**D**) = 2 cm; (**E**,**G**) = 30 μm; (**F**,**H**) = 50 μm; (**I**,**K**) = 20 μm; (**J**,**M**–**O**) = 100 μm; (**L**,**P**–**S**) = 10 μm.

8. ***Sarcoscypha longitudinalis*** M. Zeng, Q. Zhao & K.D. Hyde, sp. nov.; Figure 19

Index Fungorum number: IF 559930; Facesoffungi number: FoF 10412

Etymology: The specific epithet refers to ascospores with longitudinal striae.

Holotype: HKAS 121195

Diagnosis: This species is diagnosed by brown stipitate apothecia with villose receptacle, margin slightly wavy, or deeply split on one side, broadly fusiform ascospores with longitudinal striates.

*Saprobic* on dead wood. **Teleomorph**: *Apothecia* up to 4 cm high, 2.5 cm broad, solitary, cupulate, stipitate. *Stipe* up to 1 cm long, 5 mm broad, central, terete, solid, brown, with a thin inconspicuous hyphal pad close to base. *Receptacle* cupulate, villose, surface brown, margin slightly wavy, or deeply split on one side. *Disc* deeply concave, concolorous with the receptacle surface. *Stipal ecto-excipulum* 40–80 µm broad, composed of subhyaline to yellowish ochre, *textura porrecta*, hyphae 5–7 µm broad, hyphoid hairs abundant at the base, 4–6 µm broad, yellowish ochre. *Stipal medulla* composed of hyaline to subhyaline *textura intricata*, hyphae 3.5–6 μm broad. *Ectal excipulum* 60–100 µm broad, composed of hyaline *textura porrecta* to *textura prismatica*, brownish at the outermost part, hyphae 4–7 µm broad, mixed with 11–16 × 6–8 µm cells, with hyphoid hairs on the surface, 5–7 µm broad, septate, hyaline to subhyaline, with a rounded end. *Medullary excipulum* 235–310 µm broad, composed of hyaline to subhyaline *textura intricata*, hyphae 3–4 µm broad. *Hymenium* 280–345 µm thick, subhyaline, paraphyses slightly exceeding asci when dehydrated. *Paraphyses* 2–4 µm broad in the middle part, filiform, branched, septate, with a rounded end. *Asci* 297–359 × 12–14 µm, 8-spored, terminally operculate, subcylindrical, apex obtuse, becoming narrow towards the base. *Ascospores* [20/1/1, in H_2_O] (18.3–)19.3–21.4(–22.4) × (9.5–)10.7–12.1(–12.8) µm (Q = 1.57–1.99, **Q** = 1.79 ± 0.12), broadly fusiform, equilateral, uniseriate, multiguttulate when immature, uniguttulate when mature, ornamentation with several longitudinal striae. **Anamorph**: not seen.

Material examined: CHINA: Xishuangbanna, Bulangshan, on unidentified dead branch under broadleaved forest, 18 August 2019, Ming Zeng, ZM234 (HKAS 121195, holotype); ibid., 20 August 2019, Song Wang, ZM256 (HKAS 121196, paratype).

Notes: *Sarcoscypha* is distinguished by grey-white, yellow, orange to red apothecia which are substipitate to stipitate, glabrous to tomentose receptacle surface, ellipsoid to subcylindrical ascospores, normally with blunt ends or shallow depressions at both poles, smooth or ornamented wall, uniguttulate to multiguttulate [1,6,65,73]. Within *Sarcoscypha*, this new species is easily characterized by brown stipitate apothecia with villose receptacle, broadly fusiform, uniguttulate ascospores with longitudinal striates. According to our phylogenetic analyses, our new species is a sister group of *S. vassiljevae*. These two species share similar morphology in ascospores having a big oil drop, while *S. vassiljevae* differs from our species in grey-white hymenium and ellipsoid smooth-walled ascospores [6].

## 4. Discussion

Ekanayaka et al. [50,58] proposed the presence of ascospore sheath as a new taxonomically important character for some new and known species of *Sarcoscyphaceae*. While observing our recent collections and herbaria specimens, this particular feature appeared only in the ascospores that were treated with 5% or 10% KOH, but not in those that were mounted in water. This situation has been previously described by Pfister et al. [23] as ascospore walls loosening upon treatment with KOH, and it seems to be a universal characteristic within *Sarcoscyphaceae*. Thus, the gelatinous sheath is an invalid feature for species descriptions, much less an appropriate diagnostic feature for species identification as it is an artifact of the chemical treatment with KOH.

In this study, we introduce three new species, *N. aequispora*, *Pi. villosa* and *S. longitudinalis*, represented by two collections each based on morphology and phylogeny. In the phylogenetic analysis herein, *Phillipsia*, *Rickiella*, and *Nanoscypha* are not reciprocally monophyletic, but instead form a clade in both the ITS and combined data trees. This is a perennial unresolved problem that has also been noted in other studies [15,22]. In previous phylogenetic analyses lacking *S. vassiljevae*, *Sarcoscypha* and *Pseudopithyella* are sister taxa [15,22,23]. In studies where *S. vassiljevae* is included, *Sarcoscypha* is not monophyletic [3]. In our study, the new *Sarcoscypha* species is sister to *S. vassiljevae*; however, *Sarcoscypha* is not monophyletic in the combined gene tree. Instead, *Pseudopithyella* clustered in *Sarcoscypha* as sister to *S. coccinea*. Due to the absence of ITS data, the position of *Pseudopithyella* in the ITS tree cannot be inferred. Additional data from more *Sarcoscypha* and *Pseudopithyella* species would greatly clarify placement of these taxa.

Harrington [74] provided an ITS locus analysis and morphology of *Sarcoscypha*. In her study, *S. striatispora* clustered with two other *Nanoscypha* species, forming a distinct *Nanoscypha* clade separately from *Sarcoscypha*. Thus, Harrington [74] established the combination *N. striatispora* for *S. striatispora* W.Y. Zhuang. Furthermore, the author thought this species is more closely related to *Nanoscypha* because of its eccentrically operculate asci, and slightly equilateral ascospore with striae, which are more representative of *Nanoscypha* rather than *Sarcoscypha* [74]. The establishment of *S. striatispora* within *Sarcoscypha* was based on its distinct *textura porrecta* in the ectal excipulum [79], which differs from that of *Nanoscypha*, the latter having *textura angularis* to *textura epidermoidea* ectal excipulum [73]. Thus, Zhuang et al. [6] did not agree with this combination and proposed that the name *S. striatispora* should be retained. In the phylogeny (Figure 1), the only available *N. striatispora* strain (HMAS 61133) clusters independently from the *Sarcoscypha* species clade. Instead, *N. striatispora* groups within *Phillipsia* as sister to the clade formed by *Ph. carnicolor* Le Gal and *Ph. hydei* M. Zeng & Q. Zhao. Although there is only one strain to show the phylogenetic position of *N. striatispora*, the close relationship between *Phillipsia* and *Nanoscypha* has been demonstrated in other studies [15,22]. The relationship is also shown in the combined data tree herein, whereby *Nanoscypha* nests within *Phillipsia*. *Phillipsia* has *textura porrecta* ectal excipulum, which is similar to *N. striatispora*. Notably, the other *Nanoscypha* species represented by our new species and *N. tetraspora* also grouped within *Phillipsia*, but separately from *N. striatispora*. In terms of morphology, there are enough morphological features to distinguish *Nanoscypha* and *Philipisia*. *Nanoscypha* are often discoid to turbinate, normally less than 10 mm, centrally attached. In contrast, *Phillipsia* are often discoid, ear-shaped, and cup-shaped, normally more than 10 mm, centrally attached or eccentric [6,73,80]. The noted discrepancies warrant further exploration of the relationship between *N. striatispora* and *Phillipsia* from a phylogenetic and morphological point of view. At the moment, all *Nanoscypha* strains along with *Phillipsia* and *Rickiella* form a distinct clade, but their generic relationships remain unresolved. Additional taxa and genes will help resolve these complexities in the future.

*Cookeina* as a commonly-encountered genus of *Sarcoscyphaceae* in tropical and subtropical regions is adapted to growth in humid and hot environments [48,67]. Among the specimens we collected, a large number of species from tropical regions belong to the genus *Cookeina*, indicating a high abundance and diversity of the genus in China and Thailand. The phylogenetic position of the strains is identical in both trees. The new collections are accommodated in *C. indica*, *C. sinensis*, *C. speciosa* and *C. tricholoma*. Within *Cookeina*, the *C. speciosa* complex has high genetic variation and is divided into distinct subclades. Based on ITS and LSU phylogenies, Weinstein et al. [48] studied the correlation between colour differences of *C. speciosa* and different groups within the species complex. The colour of *C. speciosa* ranges from mauve, coral, orange, yellow to white, while there are no consistent anatomical differences among the colour variants [48]. In their study, two clades were segregated. One clade was associated with dark-coloured apothecia (mauve to deep coral), while members of the other had light-coloured apothecia (light coral, orange, yellow to white). It was then considered that the complex contains at least two taxa [48]. Taking into account our ITS inference, which contains multiple strains, their dark-coloured apothecia clade corresponds to subclades 1 and 2, while the light-coloured apothecia (light coral, orange, yellow to white) clade corresponds to subclades 3 and 4. Hence, it seems that this colour-based classification does not correspond to the phylogenetic inference herein. Six collections of *C. speciosa* cluster in a separate subclade expanding the existing diversity of the species complex. Within the *C. speciosa* complex, the placement of the sequences designated as *C. sulcipes* and *C. garethjonesii* is problematic. In our inferred phylogeny using a significantly expanded taxon sampling, neither appear as separate species from a phylogenetic point of view. Examining the type specimens and obtaining additional molecular data is necessary to disentangle this complex issue.

## 5. Conclusions

Southwestern China and Thailand are regions with high contributions to the species richness of *Sarcoscyphaceae*. Species of *Cookeina*, *Phillipsia*, and *Sarcoscypha* are very common in these areas, while *Nanoscypha* and *Pithya* have limited records. In the present study, we have redescribed five known species and established three new species in these genera. Meanwhile, *Ph. gelatinosa* is here proposed as a later epithet of *Ph. domingensis*. Our morphological and phylogenetic studies add a meaningful contribution to advancing this family toward natural classification. However, the lack of some type species and molecular data, and the presence of some species complexes, pose a challenge to future research.

## Figures and Tables

**Table 2:** Morphological characteristics of *Nanoscypha* species.

**Species**	**Apothecia**	**Hymenium**	**Excipulum**	**Asci**	**Paraphyses**	**Ascospores**	**References**
*Nanoscypha aequispora*	▸ 1–2 mm broad, scattered, shallowly cupulate when fresh, subturbinate when dry, broadly stipitate, glabrous. ▸ Stipe 400–1500 µm long, 500–2000 µm broad, funnel-shaped, wrinkled on surface, solid, cream, rarely yellowish. ▸ Receptacle shallowly concave, receptacle surface yellowish to orange, glabrous ▸ Margin undulate	▸ 280–310 µm ▸ Concave to discoid ▸ Yellow to orange	▸ Ectal: 56–94 µm, textura angularis mixed with textura prismatica, outermost layer textura porrecta ▸ Medullary: 76–192 µm, textura intricata	▸ 235–284 × 10–13 µm ▸ Cylindrical with tapering bases ▸ 8-spored ▸ Subterminally operculate	▸ 2–3 µm ▸ Filiform ▸ Septate, branched ▸ With yellowish contents	▸ 16.2–18.6 × 10.3–11.6 µm ▸ Ellipsoid, with round or slightly truncated ends ▸ Equilateral, rarely slightly inequilateral with one side flat ▸ Smooth ▸ Multiguttulate when immature, biguttulate when mature	This study
*Nanoscypha bella*	▸ 8 mm broad, discoid, orange	–	–	–	–	▸ 22.9 × 17.8 µm ▸ Ellipsoid ▸ Smooth	[76,78]
*Nanoscypha denisonii*	▸ Up to 10 mm broad, scattered, discoid, substipitate to stipitate ▸ Receptacle covered with hyaline, flexuous, septate hairs. ▸ Margin crenulate	▸ 210 µm thick ▸ Slightly convex ▸ Bright orange yellow	▸ Ectal: 35–60 µm, textura angularis, hairs originate from outer cells ▸ Medullary: 200–490 µm, textura intricata	▸ 150–215 × 10–12 µm ▸ Long cylindrical ▸ 8-spored	▸ 2.5–3.5 µm ▸ Slender, slightly enlarged at the tips	▸ 13.5–19.0 × 7–10 µm ▸ Ellipsoid to reniform ▸ Equilateral to inequilateral ▸ Smooth under light microscope, with thick longitudinal ridges and furrows under SEM ▸ Biguttulate	[75]
*Nanoscypha euspora*	▸ Up to 4 mm broad, convex to discoid, yellow	–	–	▸ 170–180 × 12 µm	▸ Filiform ▸ Hyaline	▸ 12 × 6 µm wide ▸ Rounded to ellipsoid ▸ Uniguttulate	[77]
*Nanoscypha macrospora*	▸ 2–6 mm broad, solitary to scattered, shallow cup-shaped to discoid or turbinate, stipitate to substipitate or sessile ▸ Receptacle paler, glabrous, wrinkled when dry	▸ Convex to discoid ▸ Orange to red	▸ Ectal: 20–70 µm, textura angularis, ▸ outermost layer textura epidermoidea ▸ Medullary: 40–100 µm, textura intricata	▸ 220–250 × 15–16 µm ▸ Cylindrical with long tapering bases ▸ 3- or 4-spored ▸ Eccentrically suboperculate	▸ 1–2 µm ▸ Filiform cylindrical ▸ Septate, infrequently branched	▸ 27–34 × 13–14 µm ▸ Elongate ellipsoid ▸ Equilateral, rarely inequilateral ▸ Smooth ▸ Biguttulate	[73]
*Nanoscypha pulchra*	▸ 3–5 mm broad, scattered, discoid to subturbinate, sessile to substipitate ▸ Receptacle whitish, glabrous, wrinkled when dry	▸ Concave, discoid, or slightly convex ▸ Yellow to orange	▸ Ectal: 30–80 µm, textura angularis ▸ Medullary: 120–300 µm, textura intricata	▸ 230–260 × 12–13 µm ▸ Cylindrical with tapering bases ▸ 4-, 6- or 8-spored ▸ Eccentrically suboperculate	▸ 2–3 µm ▸ Cylindrical to subcalvate, barely enlarged at their apices ▸ Septate	▸ 20–23 × 10–11 µm ▸ Ellipsoid to subreniform ▸ Inequilateral ▸ Smooth ▸ Biguttulate	[6,73]
*Nanoscypha striatispora*	▸ 6–20 mm broad, discoid to sessile to substipitate ▸ Receptacle pinkish at upper part and whitish at lower part, glabrous	▸ 180–190 µm thick ▸ Reddish orange	▸ Ectal: 40–56 µm, textura porrecta ▸ Medullary: 120–320 µm, textura intricata	▸ 160–170 × 11.7–13.0 µm ▸ Subcylindrical ▸ 8-spored ▸ Suboperculate	▸ 2 µm ▸ Subcylindrical	▸ 15.4–18.3 × 8.3–8.8 µm ▸ Ellipsoid with blunt ends ▸ Inequilateral slightly, with one side flat ▸ Minute, transverse striations on surface ▸ Biguttulate	[74,79]
*Nanoscypha tetraspora*	▸ 2–4 mm broad, scattered to crowded, shallow cup-shaped to turbinate or discoid, stipitate, substipitate, or sessile ▸ Receptacle similar in colour but paler, wrinkled when dry, glabrous ▸ Margin irregularly crenulate	▸ Concave to discoid	▸ Ectal: 20–60 µm, textura angularis, outermost layer textura epidermoidea ▸ Medullary: 80–300 µm, textura intricata	▸ 180–240 ×14–16 µm ▸ clavate ▸ 4-spored ▸ Eccentrically suboperculate	▸ 2–3 µm ▸ Filiform to cylindrical ▸ to subclavate, enlarged at their apices	▸ 18–24 × 10–12 µm ▸ Ellipsoid ▸ Inequilateral ▸ Biguttulate	[73]

**Table 3:** Sizes comparison for asci and ascospores of *Phillipsia gelatinosa* and *Phillipsia subpurpurea* (MFLU16-0612).

Species	Asci	Ascospores	References
*Phillipsia gelatinosa*	340–570 × 20–27 μm	27–36 × 14–17 μm	[58]
*Phillipsia gelatinosa* MFLU15-2360	327–392 × 11–15 µm	23.2–26.5 × 12.1–13.7 µm (Q = 1.77–2.25, **Q** = 1.93 ± 0.11)	In this study
*Phillipsia gelatinosa* MFLU 16-2956	350–380 × 12–15 µm	21.4–24.1 × 11.0–12.1 µm (Q = 1.76–2.14, **Q** = 1.97± 0.10)	In this study
*Phillipsia gelatinosa* MFLU 16-2992	359–390 × 11–15 µm	21.8–24.8 × 11.4–12.6 µm (Q = 1.71–2.15, **Q** = 1.95 ± 0.11)	In this study
*Phillipsia subpurpurea* MFLU16-0612	470–530 × 25–30 μm	30–40 × 15–20 μm	[58]
*Phillipsia subpurpurea* MFLU 16-0612	339–414 × 13–16 µm	22.1–24.7 × 12.1–14.3 µm (Q = 1.57–2.00, **Q** = 1.78 ± 0.12)	In this study

## Data Availability

Not applicable.
